# Altered splenic T-cell subpopulation dynamics in a murine model of bovine digital dermatitis-associated *Treponema* spp. infection

**DOI:** 10.3389/fimmu.2026.1744712

**Published:** 2026-07-22

**Authors:** Edeneil Jerome Valete, Myunghwan Jung, Hector Espiritu, Yong-Il Cho

**Affiliations:** 1 Department of Animal Science and Technology, Sunchon National University, Suncheon, Jeonnam, Republic of Korea; 2 Department of Microbiology, College of Medicine, Gyeongsang National University, Jinju, Gyeongnam, Republic of Korea

**Keywords:** bovine digital dermatitis, *Treponema* spp., T-cell mediated immunity, T-cell exhaustion, mouse model

## Abstract

Bovine digital dermatitis (BDD) is an infectious hoof disease associated with multiple *Treponema* species, causing significant economic and welfare concerns in the cattle industry. Studies regarding T-cell mediated immune responses are limited in BDD. Using a mouse model, this study analyzed different T-cell subsets involved in immune responses against BDD-associated treponemes. Additionally, this study measured *Treponema*-specific IgG antibody titers to elucidate humoral immune responses. Mice were experimentally infected with 2 × 10^9^ cells/mL of live *T. phagedenis* HNW1*, T. pedis* GNW45, *T. pedicariei* and their combination. After 1, 3, and 6 weeks, mice were sacrificed, blood serum was collected and T-cells were isolated from the spleen. *Treponema*-specific IgG antibody titers were measured using enzyme-linked immunosorbent assay (ELISA) and T-cell phenotypes were analyzed using flow cytometry. All *Treponema*-infected groups recorded a high *Treponema*-specific IgG antibody titers from 1 week to 6 weeks post-infection. A reduction in subpopulation percentage of CD44^+^ expressing T-cell on both CD4^+^ and CD8^+^ were observed in *Treponema*-infected groups from 1 week to 6 weeks post-infection suggesting a decrease in T-cell activation. Higher subpopulation percentage of CD44^+^, CD62L^+^ expressing T-cells on both CD4^+^ and CD8^+^ were observed in *Treponema*-infected groups at 1 week post-infection which might indicate a reduction in the activity effector T-cells, thereby decreasing their effector functions against *Treponema* infection. Higher subpopulation percentage of CD8^+^, CD44^+^ T-cells expressing IRF4^+^ and CD27^+^ without CD127 expression were observed in all *Treponema*-infected groups from 1 week to 6 weeks post-infection. These findings may be associated with altered cytotoxic T-cell differentiation and exhaustion-like T-cell features. The significantly higher subpopulation percentage of CD4^+^ T-cells expressing Foxp3^+^ compared to other CD4^+^ helper T-cell subsets suggests that *Treponema* spp. infection may be associated with a regulatory T-cell–related immune response. Our findings reveal that BDD-associated *Treponema* spp. has the potential to impair key aspects of T-cell mediated immunity as indicated by alterations in subpopulation percentage of different T-cell subsets. Collectively, our result advances our understanding of the immunological interactions between BDD-associated treponemes and the host offering valuable insights into the immunopathogenesis of BDD.

## 1 Introduction

Bovine digital dermatitis (BDD) is an infectious disease characterized by inflamed, ulcerative, and necrotizing lesions between the heel bulbs of the hind feet. It is a highly contagious disease with a poor treatment response, which has contributed to its persistence and spread to nearly all countries with dairy cattle (1). This global prevalence is a major concern in livestock farming because of the reduction in milk yield, reproductive losses, and overall decreased welfare of affected cattle (2, 3). BDD is widely recognized as having an infectious etiology involving multiple *Treponema* species (4–6). Highly motile, helical, anaerobic spirochaetes of the *Treponema* genus are the only microorganisms consistently detected within BDD lesions and are notably absent in healthy bovine foot skin tissue (7). A pilot study in South Korea revealed that Spirochaetes was the dominant phylum present in all tested BDD lesions, and *Treponema* was the most abundant genus (8). In addition, newly isolated strains, *Treponema phagedenis* HNW1 (9) *and Treponema pedis* GNW45 (10) have been described.

BDD-associated *Treponema* elicit complex host immune responses (11, 12). Despite considerable research, the interactions between BDD-associated *Treponema* and the host immune system remain poorly understood. T-cells are critical components of the host immune response against bacterial pathogens and play a key role in maintaining immune homeostasis. Their activation depends on the recognition of bacteria-derived antigens, which initiate robust biological effector mechanisms (13). Among various immune organs, the spleen, being the largest secondary lymphoid organ, serves various functions such as hematopoiesis, clearance of red blood cells, and immunological functions (14). As a specialized immune organ, the spleen plays a significant role in innate and adaptive immunity (15). Various T-cell subsets in the spleen, including CD4^+^ and CD8^+^, Foxp3^+^ expressing T-cells, and memory T-cells, may undergo dynamic changes following infection, which may reflect host-pathogen immunological interactions. Additionally, serological assays measuring specific antibodies provide a quantitative assessment of systemic immune responses. This integration could enrich our understanding of how *Treponema* spp. not only affects localized tissues but also induces systemic immune reactions.

This study presents a novel approach to elucidate the immune response following infection with BDD-associated *Treponema* spp., extending our understanding of immune dynamics beyond localized infection sites. By investigating T-cell mediated and humoral immune responses of the host, this study sheds light on how these pathogens influence immune regulation, including potential shifts in T-cell activation, exhaustion, or suppression. The observed variations over time provide deeper insights into the immunological mechanisms underlying *Treponema* spp. infection, helping to unravel its impact on host–pathogen interactions. This knowledge is crucial for identifying potential immunological biomarkers of disease progression and guiding the development of targeted therapies or vaccines aimed at mitigating the impact of BDD on animal health and welfare.

## 2 Materials and methods

### 2.1 Experimental design

Specific pathogen-free female and male mice C57BL/6J (6-weeks old) were purchased from Samtaco Co., Ltd., BIO KOREA. The animals were acclimated for 7 days before experimental infection. All experimental protocols were approved by the Gyeongsang National University (Jinju, Korea) Animal Care and Use Committee (IACUC GNU-230419-M0073). A total of 180 mice were used in this study and assigned to five treatment groups: phosphate-buffered saline (PBS) solution as the control, *T. phagedenis* HNW1 (Tpha), *T. pedis* GNW45 (Tped), a novel species *Treponema pedicariei* (Tpes), and a combination of all three *Treponema* species at equal concentrations (Tcom). Twelve (12) mice per treatment group were examined at 1, 3, and 6 weeks post-infection (**Table 1**).

**Table 1:** Experimental design used in the study showing the number of mice per treatment group across different weeks post-infection.

Treatments	Weeks post-infection		
	1-week	3-weeks	6-weeks
PBS (Control)	12	12	12
*T. phagedenis* HNW1	12	12	12
*T. pedis* GNW45	12	12	12
*Treponema pedicariei*	12	12	12
*Treponema spp.* combination	12	12	12
TOTAL	60	60	60

### 2.2 *Treponema* spp. inoculum preparation

Glycerol stocks were grown in New Oral Spirochete (NOS) medium and subcultured until the mid-exponential phase (4 days). Cultured bacteria were harvested by centrifugation at 3,000 × g for 10 min at ~4 °C. Bacterial pellets were washed and re-suspended in sterile deoxygenated phosphate-buffered saline (sdPBS) solution (Enzynomics Co., Ltd., Korea). The bacterial concentration was determined using a Petroff–Hausser counting chamber (Hausser Scientific, USA). Cell viability was determined using dark-field microscopy. To ensure bacterial viability, the inoculations were performed no more than 30 min after preparation.

### 2.3 Experimental infection of mice

Prior to inoculation, mice were anesthetized using 2,2-tribromoethanol (avertin) administered intraperitoneally at a dose of 250 mg/kg. The inoculation sites (dorsal region) were shaved and hair were completely removed using Nair^®^ (Church & Dwight Co., Inc. USA). After shaving, the inoculation sites were disinfected with 70% ethanol using cotton. The skin was pulled at the inoculation site using rubber forceps to create a tent. Mice were inoculated subcutaneously with 500 μL of live *Treponema* at a concentration of 2 × 10^9^ cells/mL using a sterile needle. For the control group, the same volume of sterile phosphate-buffered saline (sPBS) was injected. After inoculation, the mice were allowed to recover in their designated cages.

### 2.4 Spleen collection and isolation of lymphocytes

At the designated experimental time points, mice were anesthetized as described above. Once adequately anesthetized, mice were then euthanized by cervical dislocation in accordance with institutional animal care and use guidelines. The abdominal cavity was opened, and spleens were carefully excised under aseptic conditions. Spleens were immediately transferred to petri dishes containing sPBS and washed for several minutes to remove excess fat and tissue. Following the initial wash, the spleens were transferred to a new petri dish containing RPMI 1640 medium (Thermo Fisher Scientific, USA) for an additional rinse and were processed immediately. Spleen tissue was homogenized by gently pressing the spleens through a Falcon^®^ 100 μm cell strainer (Corning Life Sciences, USA) using the piston of a 1 mL syringe allowing the cells to pass through the strainer into the petri dish. The suspension was centrifuged at 350 × g for 5 min at ~4 °C, after which the supernatant was discarded. The cell pellet was re-suspended in 1 mL of 1x eBioscience™ RBC Lysis Buffer (Thermofisher Scientific, Inc., USA) and incubated on ice for 1 min to lyse red blood cells. The reaction was neutralized by adding 10 mL of RPMI 1640 medium, and the suspension was filtered through a cell strainer and centrifuged at 350 × g for 5 min at ~4 °C. The supernatants were discarded and the cell pellets were resuspended in RPMI 1640 medium.

### 2.5 Cell surface staining

Isolated cells were washed with PBS at 300–400 × g for 6 min at ~4 °C. The washed single cells were suspended at a concentration of 1.0 × 10^7^ cells/mL, and 100 µL of single cell suspensions were dispensed into each well of a 96-well plate. Prior to surface staining, cells were stained for viability with eBioscience™ Fixable Viability Dye eFluor™ 780 (Thermofisher Scientific, Inc., USA) according to the manufacturer’s instructions. Next, Fc receptors expressed on the cells were blocked using purified Rat Anti-Mouse CD16/CD32 (BD Biosciences, USA). Subsequently, the cells were stained on ice in the dark for 60 min with an antibody cocktail prepared by diluting fluorochrome-labeled antibodies targeting surface antigens in Brilliant Staining Buffer (BD Biosciences, San Jose, CA, USA), as shown in **Table 2**. The reaction was stopped by adding 150 µL of FACS buffer (PBS containing 2% bovine serum albumin and 0.1% NaN_3_) to each well, followed by three washes with 200 µL of FACS buffer.

**Table 2:** Antibody panel for flow cytometry cell surface staining.

Antibodies	Dilution ratio	Source	Catalog number
Anti-CD8a	1:100	BioLegend	100739
Anti-CD27	1:200	BD Bioscience	741518
Anti-CD127	1:250	BioLegend	135016
Anti-CD44	1:200	BD Bioscience	563114
Anti-CD62L	1:200	BD Bioscience	563252
Anti-CD4	1:250	BioLegend	100484

### 2.6 Fixation and permeabilization

One hundred (100) µL of fixation/permeabilization solution (BD Pharmingen, USA) were added to each well and incubated for 60 min on ice in the dark. The reaction was stopped by adding 100 µL of BD perm/wash™ buffer (BD Biosciences, USA) followed by subsequent washing with the same buffer at 450 × *g* for 6 min at ~4 °C. For intracellular staining, a 50 µL volume of antibody cocktail diluted in BD Perm/Wash™ buffer was added to each well (**Table 3**) and incubated on ice in the dark for 30 min. To stop the reaction, 100 μL of perm/wash™ buffer was added to each well, centrifuged and washed with 200 μL of perm/wash™ buffer. The cells were re-suspended in FACS buffer and transferred to Corning^®^ cluster tubes (Corning Life Sciences, USA) for flow cytometry analysis.

**Table 3:** Antibody panel for flow cytometric intracellular staining.

Antibodies	Dilution ratio	Source	Catalog number
Foxp3	1:50	BD Bioscience	562996
GATA3	1:50	Thermofisher	25-9966-41
T-bet	1:50	BD Bioscience	564141
RORγT	1:100	BD Bioscience	564722
IRF4	1:200	BioLegend	646408

### 2.7 Flow cytometry analysis

Data were acquired on a flow cytometer BD FACSymphony™ A3 operated with BD FACSDiva software (BD Biosciences, USA) and data analysis were performed using the FlowJo version 10.1 software (FlowJo LLC, USA). Single-stained compensation controls were acquired using the same instrument settings as the experimental samples and used to calculate the compensation matrix. Compensation was applied before final gating to correct for spectral overlap among fluorochromes. A sequential gating strategy was applied to all samples. Lymphocytes were first identified based on forward scatter area (FSC-A) and side scatter area (SSC-A) properties. Single cells were then selected by sequential singlet discrimination using FSC-H versus FSC-W, followed by SSC-A versus SSC-W. Dead cells were excluded by gating on eBioscience™ Fixable Viability Dye eFluor™ 780-negative cells (Thermo Fisher Scientific, Inc., USA). Subsequent analyses were performed on live singlet lymphocytes. CD4^+^ and CD8a^+^ lymphocyte populations were then gated and further analyzed for the subpopulation percentage of CD44^+^, CD62L^+^, CD27^+^, CD127^+^, IRF4^+^, Foxp3^+^, T-bet^+^, GATA3^+^, and RORγt^+^. Unstained controls and fluorescence minus one (FMO) controls were used to support marker-positive gate placement. Gates were applied consistently across samples within each experiment. The percentage of each reported subset was calculated as the frequency of the indicated marker-positive population within its respective parent gate. Representative gating plots are provided in **Supplementary Figure 1**. Representative FMO controls used to guide marker-positive gate placement are provided in **Supplementary Figure 2**. The denominator or parent gate used for each reported subset is summarized in **Supplementary Table 1**.

### 2.8 Preparation of *Treponema* antigens

*Treponema* spp. at the mid-exponential phase (4 days) were harvested by centrifugation at 3,000 × *g* for 10 min at ~4 °C. Approximately, 2 mL of bacterial cells were collected, washed, and re-suspended in 5 mL sterile PBS and sonicated on ice at 40% amplitude with 15 secs “on” and 59 secs “off” for 40 cycles. Cell disruption was monitored microscopically, and the process was considered complete when the suspension appeared optically clear. Samples were centrifuged at 10,000 × g for 10 min at ~4 °C. The protein concentration was determined using a Smart BCA protein assay kit (Intronbio, Korea) following the manufacturer’s instructions. Antigens were stored at –80 °C until further analysis.

### 2.9 Enzyme-linked immunosorbent assay for estimation of *Treponema*-specific IgG antibody titers

Serum samples were collected from mice via cardiac puncture. The mice were deeply anesthetized, and a 23G–25G needle attached to a 1 mL syringe was used to draw blood directly from the heart. The collected blood was immediately transferred to sterile microcentrifuge tubes and allowed to clot at room temperature for 30–60 min. Following clot formation, the samples were centrifuged at 3000 × *g* for 10 min at ~4 °C, and the serum was carefully transferred into sterile microcentrifuge tubes and stored at –80 °C until further analysis. *Treponema* antigen (10 μg protein/mL in PBS) was bound overnight to a 96-well plate (Nunc Maxisorp; Thermo Fisher Scientific, USA). Plates were washed with PBS containing 0.05% Tween-20 (PBST) (Intronbio, Korea) and then blocked with 5% skimmed milk in PBST for 1 h at 37 °C. After washing, the serum was serially diluted, added to plates, and incubated for 1 h at 37 °C. Following another washing step, horseradish peroxidase (HRP)-conjugated anti-mouse IgG (Promega, USA) was added and incubated for 1 h at 37 °C. After washing, 1-step™ Ultra TMB-ELISA substrate solution (3, 3’, 5, 5’-tetramethylbenzidine) (Thermo Fisher, Scientific, USA) was added to each well and incubated in the dark for 15 min. The reaction was then stopped with 1M sulfuric acid. Absorbance was measured at 450 nm using a microplate reader. Antibody titers were defined as the lowest dilution with an optical density equal to or greater than the mean plus two standard deviations of the wells containing only PBS (16).

### 2.10 Statistical analysis

The subpopulation percentage of each T-cell subset recorded using the FlowJo software, *Treponema*-specific IgG antibody titer (log_2_ transformation) and the mean differences in the percentages of Foxp3^+^ expressing T-cells and the Th1, Th2, and Th17 subpopulations were subjected to statistical analysis. All statistical analyses and data visualization were performed using GraphPad Prism 10.0 (GraphPad Software, La Jolla, California, USA). Group comparisons were conducted using one-way ordinary ANOVA. The means of the treatment groups were compared with that of the control group using Dunnett’s multiple comparison test. Significance is represented as *, **, ***, and **** for P < 0.05, 0.01, 0.001, and 0.0001, respectively.

## 3 Results

### 3.1 Sustained high *Treponema*-specific IgG antibody titers following *Treponema* infection

**Figure 1:**
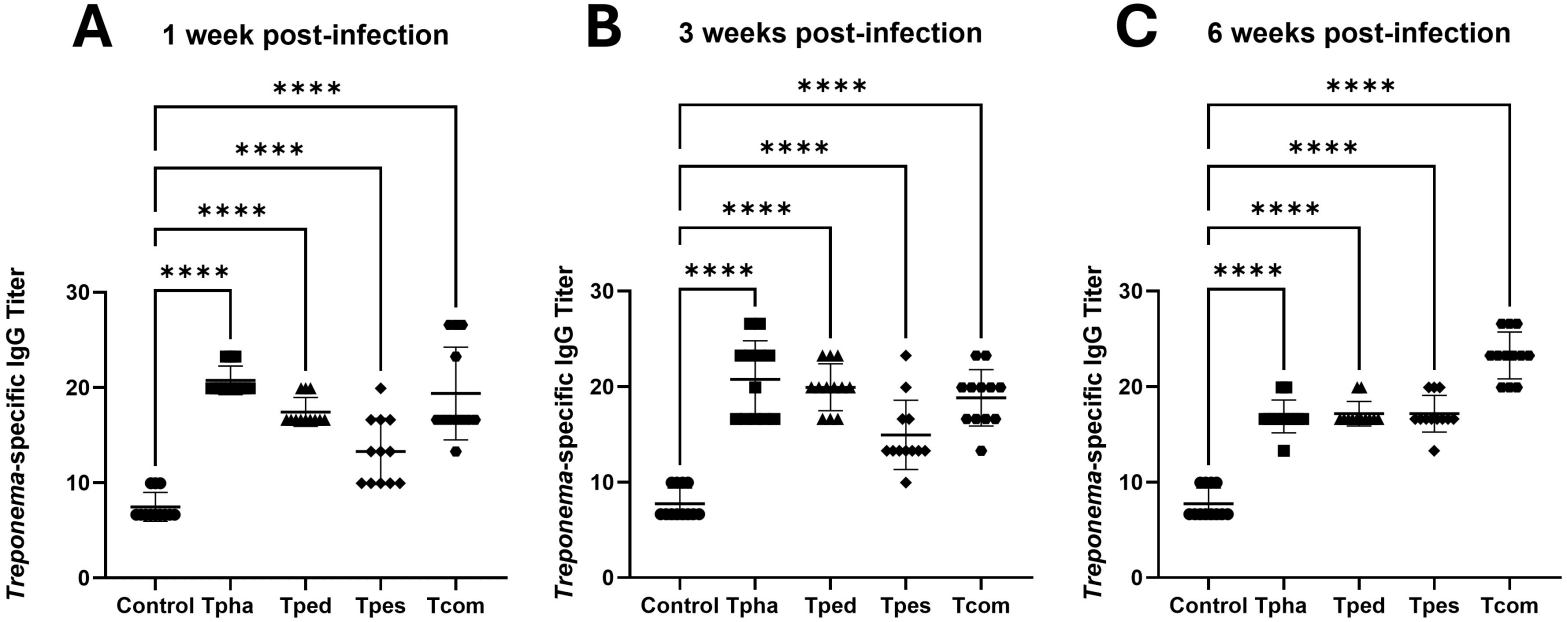
ELISA IgG titers in mice after infection with single and combination of *Treponema*. **(A)** 1 week post-infection **(B)** 3 weeks post-infection **(C)** 6 weeks post-infection. Titer were log_2_ transformed before subjecting to statistical analysis. Group comparisons were conducted using a one-way ordinary ANOVA followed by Dunnett’s multiple comparison tests for mean difference of treatment groups on the control. Significance is represented as *, **, ***, and **** for P < 0.05, 0.01, 0.001, and 0.0001, respectively. “ns” indicates results that are “not significant.” The data in each graph represent the mean ± SD.

From 1 to 6 weeks post-infection, all *Treponema-*infected groups showed significantly higher *Treponema*-specific IgG antibody titers (**Figure 1**). At 1 week post-infection, Tpha exhibited the highest IgG titer, followed by Tcom, Tped, and Tpes respectively (**Figure 1A**). At 3 weeks post-infection, Tpha continued to show the highest IgG titer, followed by Tped, Tcom, and Tpes (**Figure 1B**). By 6 weeks post-infection, the highest titer was observed in Tcom, followed by Tpha, Tped, and Tpes respectively (**Figure 1C**).

### 3.2 Persistent reduction in CD44 subpopulation percentage on CD4^+^ and CD8^+^ T-cell populations following *Treponema* infections

#### 3.2.1 CD4^+^, CD44^+^

**Figure 2:**
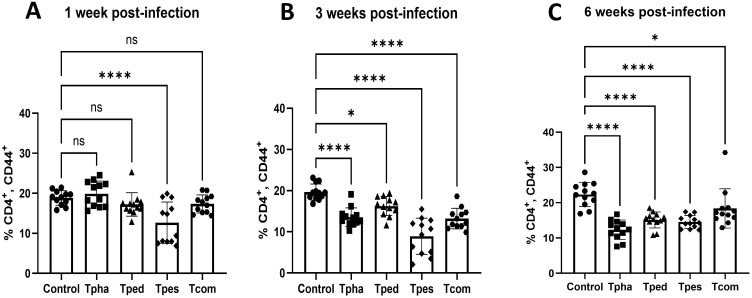
Subpopulation percentage of CD4^+^, CD44^+^ T-cells in different treatment groups. **(A)** 1 week post-infection **(B)** 3 weeks post-infection **(C)** 6 weeks post-infection. Group comparisons were conducted using a one-way ordinary ANOVA followed by Dunnett’s multiple comparison tests for mean difference of treatment groups on the control. Significance is represented as *, **, ***, and **** for P < 0.05, 0.01, 0.001, and 0.0001, respectively. “ns” indicates results that are “not significant.” The data in each graph represent the mean ± SD.

At 1 week post-infection, Tpes (12.65%) recorded a significantly lower subpopulation percentage of CD4^+^, CD44^+^ T-cells than the control (18.83%) (**Figure 2A**). At 3 weeks post-infection, all *Treponema*-infected groups exhibited a significantly lower percentage than the control group (19.66%) (**Figure 2B**). Notably, Tpes showed the lowest proportion at 8.91%, while Tped, Tpha, and Tcom showed CD4^+^, CD44^+^ subpopulations of 16.2%, 13.53%, and 13.21%, respectively. By 6 weeks post-infection, Tpha (12.33%), Tpes (14.55%), Tped (15.1%), and Tcom (18.38%) were still lower than the control (22.32%) (**Figure 2C**).

#### 3.2.2 CD8^+^, CD44^+^

**Figure 3:**
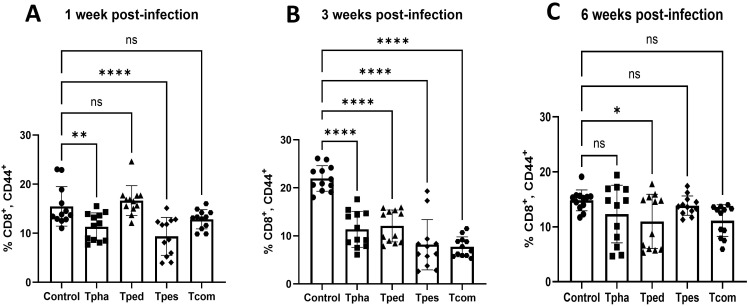
Subpopulation percentage of CD8^+^, CD44^+^ T-cells in different treatment groups. **(A)** 1 week post-infection **(B)** 3 weeks post-infection **(C)** 6 weeks post-infection. Group comparisons were conducted using a one-way ordinary ANOVA followed by Dunnett’s multiple comparison tests for mean difference of treatment groups on the control. Significance is represented as *, **, ***, and **** for P < 0.05, 0.01, 0.001, and 0.0001, respectively. “ns” indicates results that are “not significant.” The data in each graph represent the mean ± SD.

At 1 week post-infection, most of the *Treponema*-infected groups, Tcom (12.84%), Tpha (11.32%), and Tpes (9.35%), displayed significantly lower subpopulation percentages of CD8^+^, CD44^+^ T-cells than the control (17.13%) (**Figure 3A**). At 3 weeks post-infection, all *Treponema*-infected groups showed a sustained lower percentage than the control group (21.95%) (**Figure 3B**). In particular, Tcom (7.72%) and Tpes (8.16%) were the lowest among treatments, whereas Tped and Tpha recorded 12.06% and 11.37%, respectively (**Figure 3B**). At 6 weeks post-infection, Tped (10.98%) still elicited significantly lower percentages, whereas Tpes (13.84%), Tpha (12.32%), and Tcom (11.12%) showed no significant differences compared with the control (15.26%) (**Figure 3C**).

### 3.3 Temporal dynamics of CD62L^+^ subpopulation percentage after *treponema* infections

#### 3.3.1 CD4^+^, CD44^+^, CD62L^+^

**Figure 4:**
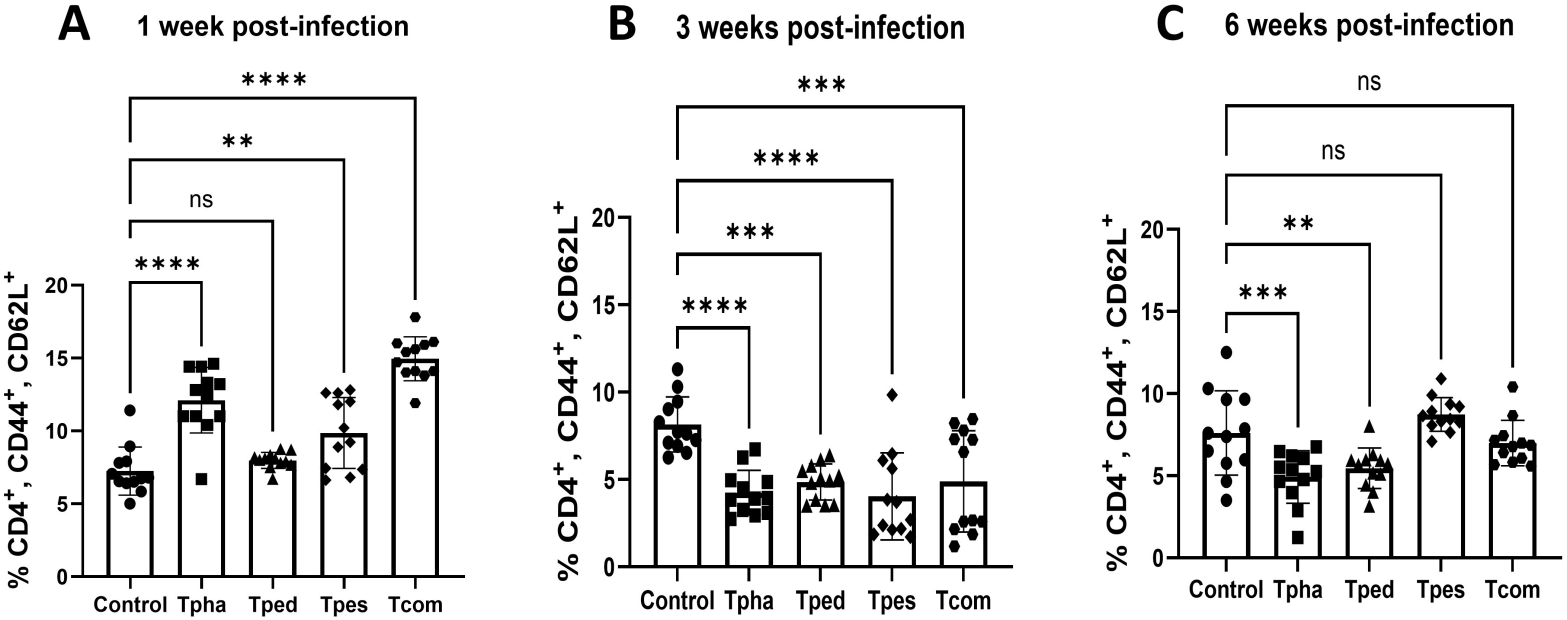
Subpopulation percentage of CD4^+^, CD44^+^, CD62L^+^ T-cells in different treatment groups. **(A)** 1 week post-infection **(B)** 3 weeks post-infection **(C)** 6 weeks post-infection. Group comparisons were conducted using a one-way ordinary ANOVA followed by Dunnett’s multiple comparison tests for mean difference of treatment groups on the control. Significance is represented as *, **, ***, and **** for P < 0.05, 0.01, 0.001, and 0.0001, respectively. “ns” indicates results that are “not significant.” The data in each graph represent the mean ± SD.

At 1 week post-infection, most of the *Treponema*-infected groups showed elevated subpopulation percentages of CD4^+^, CD44^+^, CD62L^+^ subsets compared to the control (7.24%) (**Figure 4A**). The highest subpopulation percentage was recorded by Tcom (14.95%), followed by Tpha (12.11%) and Tpes (9.86%) (**Figure 4A**). Three weeks post-infection, all *Treponema*-infected groups showed a significantly lower subpopulation percentage ranging from 3.87–5.62% compared to the control (8.15%) (**Figure 4B**). By 6 weeks post-infection, Tpha (4.93%) and Tped (5.45%) infected group still shows a lower subpopulation percentage, whereas Tpes (8.73%) and Tcom (6.99%) showed subpopulation percentages comparable to those of the control (7.61%) (**Figure 4C**).

#### 3.3.2 CD8^+^, CD44^+^, CD62L^+^

**Figure 5:**
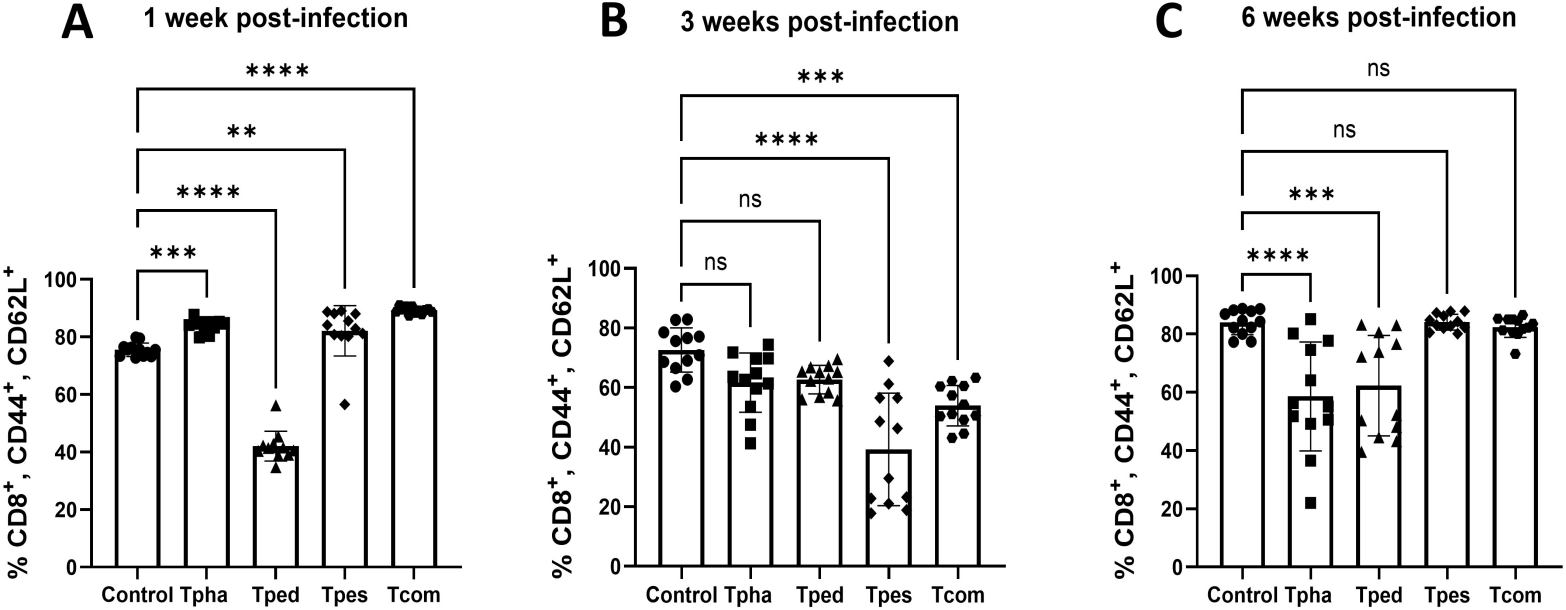
Subpopulation percentage of CD8^+^, CD44^+^, CD62L^+^ T-cells in different treatment groups. **(A)** 1 week post-infection **(B)** 3 weeks post-infection **(C)** 6 weeks post-infection. Group comparisons were conducted using a one-way ordinary ANOVA followed by Dunnett’s multiple comparison tests for mean difference of treatment groups on the control. Significance is represented as *, **, ***, and **** for P < 0.05, 0.01, 0.001, and 0.0001, respectively. “ns” indicates results that are “not significant.” The data in each graph represent the mean ± SD.

At 1 week post-infection, most of the *Treponema*-infected group showed a significantly higher subpopulation percentage than the control (74.79%), in which Tcom showed the highest percentage of subpopulation (89.33%), followed by Tpha (84.00%) and Tpes (82.12%). In contrast, Tped showed a significantly lower percentage among all the treatments (**Figure 5A**). At 3 weeks post-infection, all *Treponema*-infected groups showed a lower percentage of subpopulations than the control, in which *Tpes* recorded the lowest percentage (39.27%) (**Figure 5B**). Other *Treponema*-infected groups also showed significantly lower populations, with subpopulation percentages ranging from 53.92% to 61.68% (**Figure 5B**). At 6 weeks post-infection, a significantly lower percentage was observed in Tpha (58.59%) and Tped (62.28%) than in the control (84.03%) (**Figure 5C**).

### 3.4 Sustained high CD8^+^, CD44^+^, CD27^+^, CD127^-^ T-cell subpopulation following *Treponema* infection

**Figure 6:**
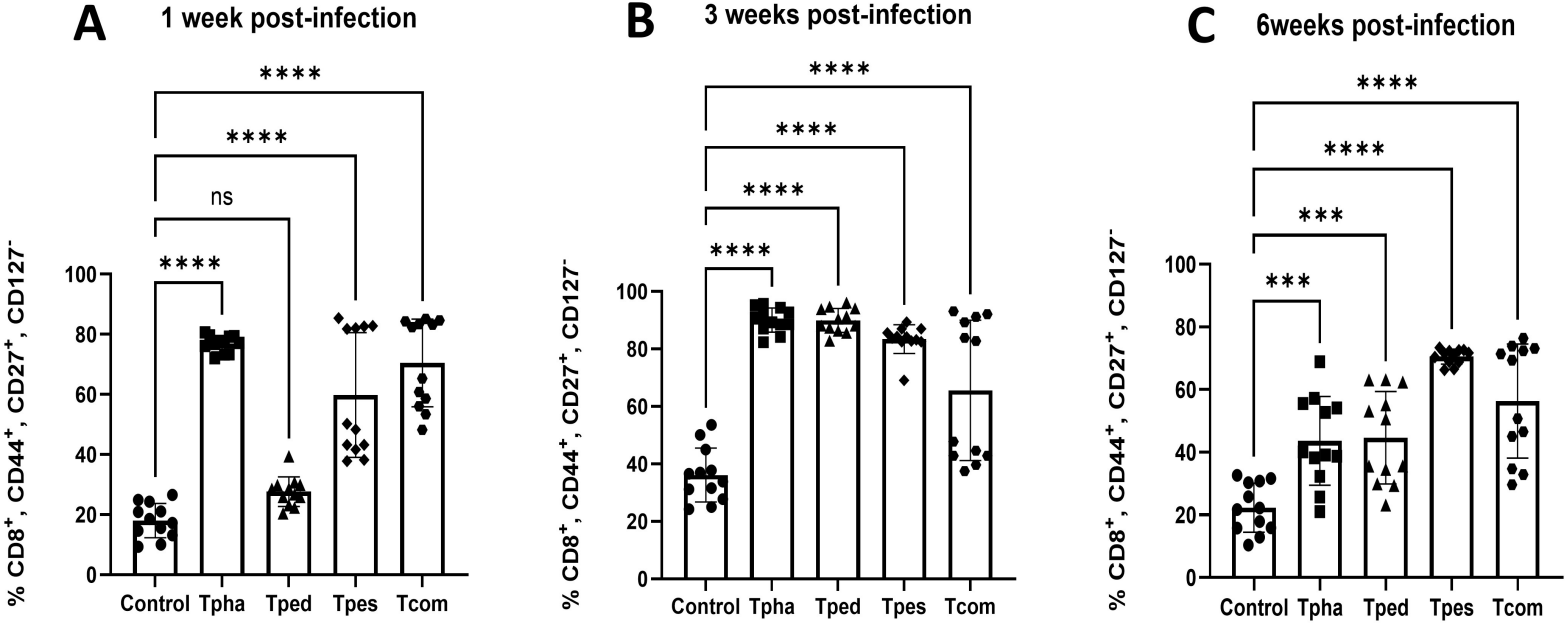
Subpopulation percentage of CD8^+^, CD44^+^, CD27^+^, CD127^-^ T-cells in different treatment groups. **(A)** 1 week post-infection **(B)** 3 weeks post-infection **(C)** 6 weeks post-infection. Group comparisons were conducted using a one-way ordinary ANOVA followed by Dunnett’s multiple comparison tests for mean difference of treatment groups on the control. Significance is represented as *, **, ***, and **** for P < 0.05, 0.01, 0.001, and 0.0001, respectively. “ns” indicates results that are “not significant.” The data in each graph represent the mean ± SD.

At 1 week post-infection, *Treponema*-infected groups exhibited a significantly higher subpopulation percentage than the control (17.98%), in which Tpha (76.9%) displayed the highest population percentage, followed by Tcom (70.44%) and Tpes (59.76%) (**Figure 6A**). At 3 weeks post-infection, all *Treponema*-infected groups still exhibited significantly higher population percentages compared to the control group (36.16%). Among these, Tpha showed the highest percentage (90.08%), followed by Tped (89.93%), Tpes (83.42%), and Tcom (65.65%) (**Figure 6B**). At 6 weeks post-infection, the population percentages in all *Treponema*-infected groups remained significantly elevated relative to the control (22.33%). At this time point, Tpes displayed the highest population percentage (70.51%), followed by Tcom (56.33%), Tped (44.61%), and Tpha (43.63%) (**Figure 6C**).

### 3.5 Formation of the CD4^+^ helper T-cell subpopulation following *Treponema* infection

**Figure 7:**
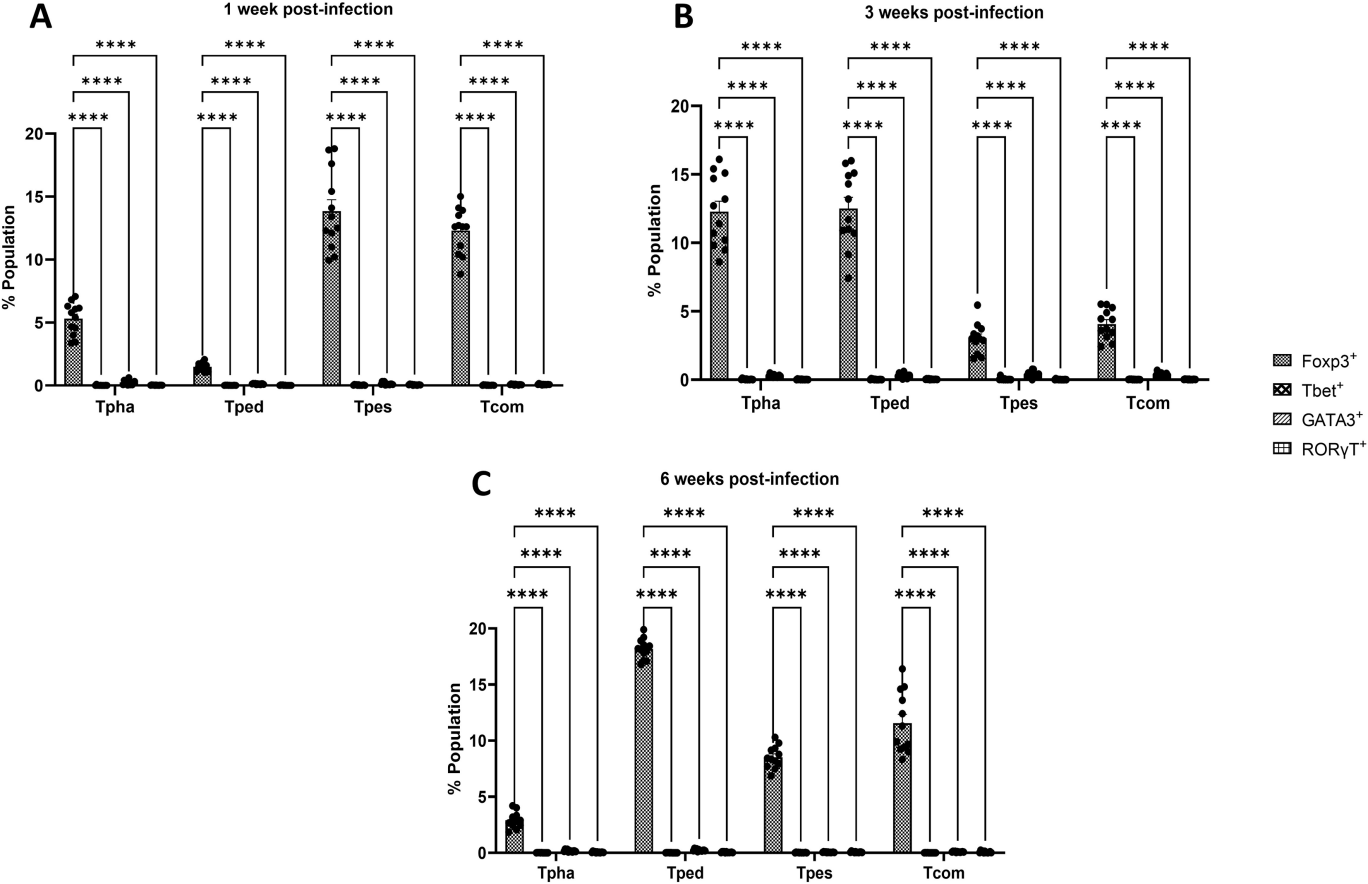
Percentage of CD4^+^ helper T-cell subpopulations across different treatment groups and weeks post-infection. **(A)** 1 week post-infection **(B)** 3 weeks post-infection **(C)** 6 weeks post-infection. Group comparisons were conducted using a one-way ordinary ANOVA followed by Dunnett’s multiple comparison tests for mean difference of different CD4^+^ helper T-cell subpopulation. Significance is represented as *, **, ***, and **** for P < 0.05, 0.01, 0.001, and 0.0001, respectively. “ns” indicates results that are “not significant.” The data in each graph represent the mean ± SD.

In this study, significant Foxp3^+^ regulatory-phenotype T-cells activation was only observed in the CD4^+^ helper T-cell subpopulation (**Figure 7**) in all *Treponema*-infected groups. At 1 week post-infection, the highest percentage of Foxp3^+^ regulatory-phenotype T-cells was detected in Tpes (13.84%), followed by Tcom (12.29%), Tpha (5.30%), and Tped (1.48%) (**Figure 7A**). By 3 weeks post-infection, Tped exhibited the highest percentage (12.51%), followed by Tcom (4.07%), Tpes (3.04%), and Tpha (2.92%) (**Figure 7B**). At 6 weeks post-infection, the highest percentage was observed in the Tpha (12.28%), followed by Tcom (11.56%), Tped (10.09%), and Tpes (8.53%) (**Figure 7C**).

### 3.6 Sustained high CD8^+^, CD44^+^, IRF4^+^ T-cell subpopulation following *Treponema* infection

**Figure 8:**
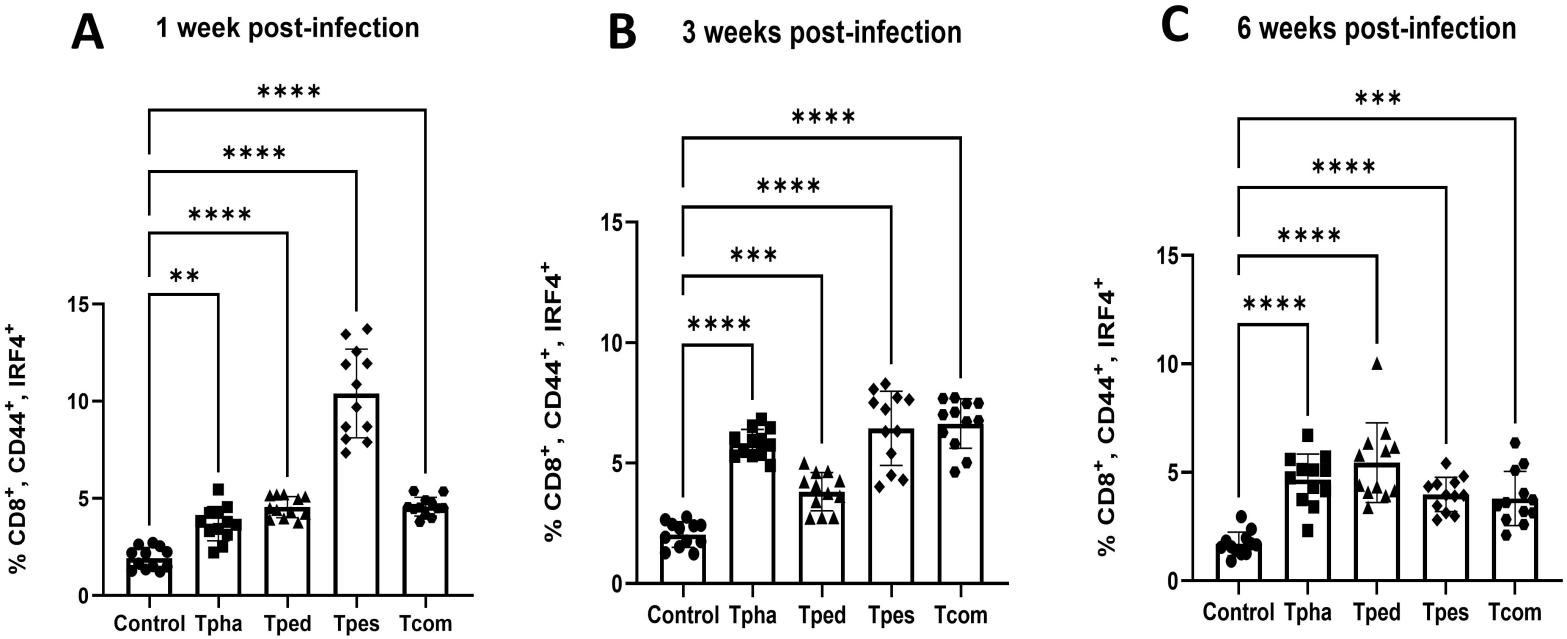
Subpopulation percentage of CD8^+^, CD44^+^, IRF4^+^ T-cells in different treatment groups. **(A)** 1 week post-infection **(B)** 3 weeks post-infection **(C)** 6 weeks post-infection. Group comparisons were conducted using a one-way ordinary ANOVA followed by Dunnett’s multiple comparison tests for mean difference of treatment groups on the control. Significance is represented as *, **, ***, and **** for P < 0.05, 0.01, 0.001, and 0.0001, respectively. “ns” indicates results that are “not significant.” The data in each graph represent the mean ± SD.

At 1 week post-infection, a significantly higher subpopulation percentage compared to the control (1.92%) was observed in *Treponema*-infected groups. Highest percentage was recorded by Tpes (10.40%), followed by Tcom (4.55%), Tped (4.55%) and Tpha (3.70%) (**Figure 8A**). At weeks 3 post-infection, all *Treponema*-infected groups exhibited significantly higher population percentages compared to the control group (2.03%). Tcom recorded the highest percentage (6.64%), followed by Tpes (6.44%), Tpha (5.82%) and Tped (3.82%) (**Figure 8B**). At 6 weeks post-infection, the population percentages in all *Treponema*-infected groups remained significantly elevated compared to the control (1.70%). Highest percentage was observed in Tped (5.44%), followed by Tpha (4.67%), Tpes (3.98%) and Tcom (3.79%) (**Figure 8C**).

## 4 Discussion

### 4.1 *Treponema*-specific IgG antibody titer

Our study showed that *Treponema* infection could sustain high *Treponema*-specific IgG antibody titers for up to 6 weeks post-infection, indicating a prolonged systemic immune response. The IgG response after experimental infection in mice suggests that *Treponema* inoculated subcutaneously was exposed to systemic immunity, thereby triggering systemic immune responses. Strong serum antibody responses have been elicited against *Treponema* antigens derived from lesions in cattle with digital dermatitis (11). In calf models of infection, a robust antibody response to treponemal antigens is observed, even within a 4-week period (17).

### 4.2 CD44^+^

CD44 is a well-known surface molecule that plays a critical role in T-cell activation and effector function (18). Its expression is markedly upregulated following antigenic stimulation, serving as marker of T-cell activation (19–21). The absence or dysfunction of CD44 has been shown to impair T-cell activation and cytokine production, leading to a weakened overall immune response (22). In the present study, we observed a significant reduction in the percentages of CD4^+^ and CD8^+^ T-cell subpopulations expressing CD44^+^ as early as 1 week post-infection, which persisted through week six in *Treponema*-infected groups. This pattern contrasts with the typical response observed in other bacterial infections, in which antigen exposure strongly induces CD44 expression as part of T-cell activation and effector differentiation. The sustained decrease in CD44^+^ T-cell subpopulations therefore suggests that *Treponema* infection does not effectively elicit conventional T-cell activation and may disrupt host immune signaling pathways. One possible explanation is that *Treponema* infection may interfere with CD44-associated immune signaling. CD44 is not only an activation marker but also a receptor for hyaluronic acid (HA), a major extracellular matrix component (23). Mechanistically, CD44-HA signaling has been documented to induces a positive feedback loop that stabilizes and up-regulates CD44 expression on activated T-cells during inflammation and facilitate, homing, rolling, and extravasation of lymphocytes into inflammatory sites (18, 24, 25). Therefore, disruption of this axis may potentially impair T-cell activation, migration, and CD44-mediated costimulatory signals during infection. In other spirochaetal infection, *Treponema pallidum* contain hyaluronidase that disrupts HA facilitating its invasiveness and ability to penetrate tissues (26). This HA disruption by *Treponema*-derived hyaluronidase may weaken CD44-mediated signaling/trafficking and contributes to suboptimal expansion, migration, or persistence of CD44-expressing activated T-cell subsets. It is possible that *Treponema* causing BDD might share similar mechanisms either through genes homologous to known hyaluronidases or through unique ECM-degrading enzymes that could affect CD44-HA signaling possibly leading to a reduction in the immune response responsible for clearing *Treponema* which might be a key mechanism involved in the establishment of chronic infection. However, we did not directly measure hyaluronan abundance, hyaluronidase activity, CD44 transcription, or CD44 ligand binding. Therefore, we cannot conclude that degradation of HA directly caused reduced CD44 expression on T-cells. Other factors might contribute to the reduced CD44 expression on T-cells like altered activation status, impaired antigen-driven activation, changes in T-cell differentiation, or altered trafficking.

### 4.3 CD62L^+^

CD62L is classically recognized as both an adhesion molecule and a lymph node–homing receptor, facilitating T-cell trafficking to secondary lymphoid organs and enabling their retention or return to these sites (27, 28). It plays a pivotal role in delineating T-cell subsets, especially distinguishing naive and central memory (TCM) from their effector cells (29, 30). However, the functional identity of these subsets is context-dependent, being shaped by antigen persistence, tissue environment, and cytokine cues (31). Typically, within a week following infection with a bacterial pathogen, T-cells differentiate into effector cells that downregulate CD62L and migrate from lymphoid tissues to peripheral sites of infection. The transition from a CD44^+^, CD62L^+^ to a CD44^+^, CD62L^-^ phenotype reflects their shift from a lymph node–homing state to an effector state capable of executing immune functions in peripheral tissues (32, 33). Following pathogen clearance, a subset of these effector cells re-express high levels of CD62L and acquire central-memory characteristics, marking the establishment of a resolved and durable immune response (29). The increased subpopulation of CD44^+^, CD62L^+^ T-cells at 1 week post-infection in our study may reflect a skewing of differentiation away from robust effector phenotypes toward dysfunctional or ‘memory-like’ cells unable to exert full effector function. These cell subpopulations suggest that, although antigen recognition and T-cell activation occurred, these cells may not have efficiently transitioned into migratory effector populations. Although CD62L^+^ T-cell responses were altered across *Treponema*-infected groups, some inter-group variation was observed, particularly at 1 week post-infection. The higher proportion of CD4^+^, CD44, ^+^CD62L^+^ T-cells in the Tcom group may suggest that combined *Treponema* exposure induces a distinct early systemic T-cell response. This observation may be relevant because natural BDD lesions are commonly associated with polymicrobial *Treponema* communities rather than a single *Treponema* species (7, 34). In contrast, the single-species infection groups may provide useful exploratory insight into species-associated immune response patterns. However, this finding should be interpreted as exploratory, as the present study was not designed to directly rank the pathogenicity of individual *Treponema* species or to correlate CD62L expression patterns with BDD lesion severity. *Treponema* spp. are well known for inducing chronic infections characterized by immune evasion and persistent antigenic stimulation (35, 36). Such phenomenon has been reported in other chronic infections, where prolonged antigen exposure and sustained inflammatory signaling restrain terminal effector differentiation and promote the accumulation of transitional or exhausted T-cells (37, 38). Similar patterns have also been described in persistent infections caused by *Listeria monocytogenes*, *Mycobacterium tuberculosis*, and lymphocytic choriomeningitis virus (LCMV), in which CD62L^+^ T-cells emerge early but fail to mature into fully functional effector or memory subsets under continuous antigenic stimulation (39). This interpretation is further supported by the sustained increase in the CD8^+^, CD44^+^, CD27^+^ splenic subpopulation observed at 1, 3, and 6 weeks post-infection.

### 4.4 CD27^+^

CD27 belongs to the tumor necrosis factor (TNF) receptor family, which is involved in the activation and differentiation of immune cells (40). Studies have revealed that the interaction between CD27 and its cellular ligand CD70 controls the formation of effector and memory T-cells (41, 42). The observed increase in CD8^+^, CD44^+^, CD27^+^ subpopulation percentages in our study could be associated with the development of memory T-cells during infection. However, in the subpopulations of CD8^+^, CD44^+^, CD27^+^ splenocytes, CD127 expression was not observed at all in this study. Essentially, CD127 promotes the survival and maintenance of memory T-cell pools and without CD127 expression, memory formation by T-cells is impaired (43). Usually, CD127 expression during the acute phase of infection indicates a subset of effector cells that can persist during the memory phase (44). CD127 is also essential for CD8^+^ T-cell proliferation and function, as well as for the formation of memory T-cells (45). The absence of CD127 expression provides evidence against the idea that CD8^+^, CD44^+^, CD27^+^, subpopulations are associated with the formation of central memory cells following *Treponema* infection. Additionally, the increased expression of CD62L^+^ observed after infection may not necessarily indicate its involvement in central memory cell formation. Numerous studies have reported that sustained expression of CD27 following infection is associated with T-cell exhaustion in environments with persistent antigen exposure (46, 47), which may be closely associated with the chronic nature of *Treponema* spp. infection. During clonal expansion and normal effector cell differentiation, CD27 expression typically decreases (48). Interestingly, the *Treponema*-infected groups in this study exhibited a high percentage of CD8^+^, CD44^+^, CD27^+^ T-cells lacking CD127 expression, which persisted up to 6 weeks post-infection. This observation may be associated with potentially impaired effector formation of cytotoxic T-cells (CTLs). Persistent viral antigen load suppresses CD127 expression in effector T-cells and correlates with the exhaustion of a previously stable effector T-cell population (49). Accordingly, persistent CD27 expression may be correlated with the decreased effector function of CTLs (48). It is believed that prolonged CD27 signaling also leads to T-cell exhaustion (50) and can cause T-cell dysfunction (51). Uncontrolled CD27 co-stimulation deregulates effector T cell differentiation, promotes exhaustion, and contributes to detrimental outcomes in chronic infections (52). Considering these prior findings, the sustain expression of molecules typically associated with central memory cells, such as CD27 in response to *Treponema* spp. infection may reflect an exhaustion-like phenotype state of T-cell rather than memory cell differentiation.

### 4.5 Forkhead box protein P3

Regulatory T-cells (Tregs) expressing the Foxp3 transcription factor are crucial regulators of immunological self-tolerance and homeostasis (53, 54). Foxp3 regulate inflammation in response to infectious pathogens (55). These mechanisms employed by Foxp3^+^ expressing T-cells are also utilized by numerous pathogens that can chronically persist in the host. During the coevolution of microbial pathogens and the host immune system, pathogens develop sophisticated strategies to evade host defense by taking advantage of the regulatory functions of Foxp3 (56). In syphilis caused by *T. pallidum*, an increase in Foxp3^+^ regulatory-phenotype T-cells activity initially reduces tissue damage, but may also promote bacterial survival in the later stages of the infection, contributing to chronic and latent disease states (57). *Helicobacter pylori* also mediates the skewing of the T-cell response towards Foxp3^+^ regulatory-phenotype T-cells, which increases the bacterial load and facilitates the chronicity of the infection by suppressing protective immune responses (58). *Brucella* spp. also possesses mechanisms that can downregulate immune responses by inducing Foxp3^+^ regulatory-phenotype T-cells (59). In the present study, *Treponema* infection was associated with an increased percentage of Foxp3^+^ cells within the CD4^+^ T-cell population compared with other helper T-cell subpopulations. This finding suggests that BDD-associated *Treponema* spp. may be linked to a Treg–associated immune response.

### 4.6 Interferon regulatory factor 4

The transcription factor IRF4 is a central regulator of T cell fate and function, integrating signals from the TCR to dictate effector differentiation, metabolic activity, and memory potential (60, 61). In particular, IRF4 expression levels are tightly coupled to TCR signal strength and persistence, with transient antigen stimulation favoring short-term effector responses, and sustained antigen exposure driving prolonged IRF4 expression (62). In our study, we observed a sustained upregulation of CD8^+^, CD44^+^, IRF4^+^, T-cells from 1 week through 6 weeks post-infection in *Treponema*-infected groups. This prolonged elevation suggests that *Treponema* infection might cause persistent antigenic stimulation, which may continuously engage TCR, thereby maintaining IRF4 expression. This is consistent with the biology of *Treponema* spp., which is known to cause chronic infections, immune evasion, and tissue persistence in BDD (1). While IRF4 is important for effector expansion and the cytotoxic activity of CD8^+^ T-cells, its sustained high-level expression is also linked to the initiation of exhaustion programs in T-cells (63, 64). Indeed, exhausted T-cells, compared to functional memory or effector T-cells, express elevated IRF4, upregulate inhibitory receptors like PD-1, LAG-3, TIM-3 and progressively lose cytokine production and proliferative capacity (65). Thus, the persistent CD8^+^, CD44^+^, IRF4^+^ T-cell population observed in our study may represent an exhaustion-like phenotype. These results align with the observed increase in Foxp3^+^ regulatory-phenotype T-cells following *Treponema* spp. infection in the present study. It has been found that high IRF4 expression are positively correlated with Foxp3 expression (66) and IRF4 favors the development and suppressive activity of Foxp3^+^ regulatory-phenotype T-cells (67). Together, our findings suggest that *Treponema* infection may induce an exhaustion-like pattern in T-cells, a phenomenon well described in chronic viral and tumor models but underexplored in chronic bacterial infections. Such mechanism might provide a possible explanation for the chronicity and recurrence of BDD lesions, as impaired CD8 T-cell responses would limit the effective clearance of *Treponema* spp. Moreover, this aligns with broader concepts of chronic bacterial infections (e.g., *Mycobacterium tuberculosis*), in which persistent antigen exposure drive dysfunctional T-cell states (64).

Further investigation of how *Treponema* spp. manipulates host immune pathways at the molecular level will provide crucial insights into the chronic nature of BDD. Evidence from experimental syphilis models shows that dendritic cell activation by *T. pallidum* is markedly delayed because its lipoproteins are not surface-exposed and require bacterial degradation prior to immune recognition (68). This delayed innate activation mirrors the mechanisms observed in *M. tuberculosis* infection where they inhibit apoptosis and hinder dendritic cell migration, ultimately prolonging the onset of T-cell priming (69). By employing comparable immune evasion strategies, BDD-associated *Treponema* may evade early immune surveillance, thereby favoring persistence within host tissues. Rather than resolving the infection, these skewed responses contribute to T-cell exhaustion and defective effector differentiation, which are the hallmarks of chronic spirochetal infections (36). Our findings also suggest a potential association between *Treponema* spp. infection and increased Foxp3^+^ T-cell frequencies. Further investigation into how *Treponema* spp. infection influences Treg-associated immune regulation may provide additional insights into the immunological processes contributing to the chronic nature of BDD.

Although this study provides evidence that BDD-associated *Treponema* spp. can induce changes in T-cell subpopulation percentage and *Treponema*-specific IgG responses in a mouse infection model, several limitations should be acknowledged. First, BDD is a localized hoof-skin disease in cattle while the present study used dorsal subcutaneous inoculation in mice. Therefore, this model does not fully reproduce the anatomical structure, local hoof-skin environment, polymicrobial nature, mechanical stress, moisture, and chronic lesion conditions associated with natural BDD. Second, our analysis focused mainly on splenic T-cell populations and serum antibody responses, therefore, the observed changes should be interpreted as systemic immune alterations rather than direct evidence of immune events occurring within bovine hoof lesions. Future studies in bovine hoof-skin infection models or naturally occurring BDD cases should integrate lesion stage, local cytokine profiles, *Treponema* burden, and tissue-resident immune-cell phenotyping. Third, although the observed changes in CD44^+^, CD62L^+^, CD27^+^, Foxp3^+^, and IRF4^+^ T-cell populations suggest alterations in T-cell activation, differentiation, and immune regulation following *Treponema* infection, the present study primarily evaluated phenotypic changes and did not directly assess T-cell effector function. Therefore, these findings should be interpreted as immunological associations rather than definitive evidence of impaired T-cell function or exhaustion. Future studies incorporating antigen-specific ex vivo restimulation, PD-1 expression, cytokine production, proliferative capacity, cytotoxic activity, and Treg functional assays would provide further insight into the biological significance of these altered T-cell phenotypes. In addition, although potential mechanisms involving CD44-associated pathways were discussed, the underlying molecular mechanisms were not directly investigated in the present study. Further studies examining host–pathogen interactions affecting CD44-associated signaling and T-cell responses may help clarify the mechanisms underlying *Treponema*-associated immune modulation. Collectively, these observations highlight that BDD pathogenesis is closely tied to the capacity of *Treponema* spp. to induce potentially impaired and exhaustive-like T-cell responses, offering insights for disease chronicity and a potential target for therapeutic intervention.

## 5 Conclusions

This study demonstrated host T-cell immune responses following experimental infection with BDD-associated *Treponema* highlighting novel insights that may contribute to immune dysregulation and bacterial persistence. Notably, *Treponema* infection led to a reduction in CD44 expression, which might indicate decrease in T-cell activation. Increased expression of CD62L may reduce the activity of effector T-cells, thereby decreasing their effector functions against *Treponema* spp. infection. Furthermore, only subpopulation of Foxp3^+^ regulatory-phenotype T-cells was observed among the CD4^+^ T-cell subpopulation, suggesting that *Treponema* spp. infection may be associated with a Treg related immune response. Additionally, sustained expression of CD27 may be interpreted as a sign of T-cell activation, however, the near absence of CD27^+^, CD127^+^ double-positive cells suggests that CD27 expression is more likely associated with potentially impaired effector T-cells or exhaustion-like phenotype following infection. In this experiment, the observed low expression of CD44 following *Treponema* spp. infection, initially high expression of CD62L, sustained high expression of CD27 along with low levels of CD127, sustained high expression of IRF4 collectively suggest characteristics consistent with potentially altered or impaired of T-cell activation induced by *Treponema* spp. infection. These findings are consistent with the elevated levels of Foxp3^+^ regulatory-phenotype T-cells subpopulation observed in this study. Collectively, these immunological changes suggest that *Treponema* spp. potentially impairs key aspects of T-cell-mediated immunity, which might contribute to bacterial persistence and chronic nature of BDD. Hence, these findings advances our understanding of the immunopathogenesis of BDD and provide a valuable insight into the immunological interactions between the host and BDD-associated treponemes.

## Data Availability

The raw data supporting the conclusions of this article will be made available by the authors, without undue reservation.

## References

[1] Evans NJ Murray RD Carter SD . Bovine digital dermatitis: Current concepts from laboratory to farm. Vet J. (2016) 211:3–13. doi: 10.1016/j.tvjl.2015.10.028 27061657

[2] Wilson-Welder J Alt D Nally J . Digital dermatitis in cattle: Current bacterial and immunological findings. Animals. (2015) 5:1114–35. doi: 10.3390/ani5040400 26569318 PMC4693204

[3] Omontese BO Bellet-Elias R Molinero A Catandi GD Casagrande R Rodriguez Z . Association between hoof lesions and fertility in lactating Jersey cows. J Dairy Sci. (2020) 103:3401–13. doi: 10.3168/jds.2019-17252 32057429

[4] Evans NJ Brown JM Demirkan I Murray RD Vink WD Blowey RW . Three unique groups of spirochetes isolated from digital dermatitis lesions in UK cattle. Vet Microbiol. (2008) 130:141–50. doi: 10.1016/j.vetmic.2007.12.019 18243592

[5] Klitgaard K Foix Bretó A Boye M Jensen TK . Targeting the Treponemal microbiome of digital dermatitis infections by high-resolution phylogenetic analyses and comparison with fluorescent In Situ hybridization. J Clin Microbiol. (2013) 51:2212–9. doi: 10.1128/JCM.00320-13 23658264 PMC3697659

[6] Zinicola M Higgins H Lima S MaChado V Guard C Bicalho R . Shotgun metagenomic sequencing reveals functional genes and microbiome associated with bovine digital dermatitis. PloS One. (2015) 10:e0133674. doi: 10.1371/journal.pone.0133674 26193110 PMC4508036

[7] Evans NJ Brown JM Demirkan I Singh P Getty B Timofte D . Association of unique, isolated Treponemes with bovine digital dermatitis lesions. J Clin Microbiol. (2009) 47:689–96. doi: 10.1128/JCM.01914-08 19144804 PMC2650952

[8] Mamuad LL Seo BJ Faruk M Espiritu HM Jin SJ Kim WI . Treponema spp. the dominant pathogen in the lesion of bovine digital dermatitis and its characterization in dairy cattle. Vet Microbiol. (2020) 245:108696. doi: 10.1016/j.vetmic.2020.108696 32456812

[9] Espiritu HM Mamuad LL Jin S Kim S Lee S Cho Y . High quality genome sequence of Treponema phagedenis KS1 isolated from bovine digital dermatitis. J Anim Sci Technol. (2020) 62:948–51. doi: 10.5187/jast.2020.62.6.948 33987574 PMC7721586

[10] Espiritu H Mamuad L Valete EJ Lee SS Cho YI . Complete genome sequence of Treponema pedis GNW45 isolated from dairy cattle with active bovine digital dermatitis in Korea. J Anim Sci Technol. (2024) 66:1079–82. doi: 10.5187/jast.2023.e61 39398312 PMC11466731

[11] Moe KK Yano T Misumi K Kubota C Yamazaki W Muguruma M . Analysis of the IgG immune response to Treponema phagedenis -like spirochetes in individual dairy cattle with papillomatous digital dermatitis. Clin Vaccine Immunol. (2010) 17:376–83. doi: 10.1128/CVI.00464-09 20107009 PMC2837959

[12] Watts KM Fodor C Beninger C Lahiri P Arrazuria R De Buck J . A differential innate immune response in active and chronic stages of bovine infectious digital dermatitis. Front Microbiol. (2018) 9:1586. doi: 10.3389/fmicb.2018.01586 30072966 PMC6060252

[13] Shepherd FR McLaren JE . T cell immunity to bacterial pathogens: Mechanisms of immune control and bacterial evasion. Int J Mol Sci. (2020) 21:6144. doi: 10.3390/ijms21176144 32858901 PMC7504484

[14] Lewis SM Williams A Eisenbarth SC . Structure and function of the immune system in the spleen. Sci Immunol. (2019) 4:eaau6085. doi: 10.1126/sciimmunol.aau6085 30824527 PMC6495537

[15] Aliyu M Zohora F Akbar Saboor-Yaraghi A Department of Immunology, School of Public Health, Tehran University of Medical Sciences, International Campus, TUMS-IC, Tehran, Iran Department of Medical Microbiology, Faculty of Clinical Science, College of Health Sciences, Bayero University, Kano, Nigeria Department of Pathology, Faculty of Veterinary Science, Bangladesh Agricultural University, Mymensingh-2202, Dhaka, Bangladesh . Spleen in innate and adaptive immunity regulation. AIMS Allergy Immunol. (2021) 5:1–17. doi: 10.3934/Allergy.2021001

[16] Wilson-Welder JH Nally JE Alt DP Palmer MV Coatney J Plummer P . Experimental Transmission of Bovine Digital Dermatitis to Sheep: Development of an Infection Model. Vet Pathol.. (2018) 55:245–57. doi: 10.1177/0300985817736572 29145798

[17] Coatney JW Krull AC Gorden PJ Shearer J Humphrey S Olsen S . Assessment of immunological response to digital dermatitis pathogen derived antigens following infection, recovery, and reinfection. Front Vet Sci. (2024) 11:1487316. doi: 10.3389/fvets.2024.1487316 39711796 PMC11660806

[18] Baaten BJG Tinoco R Chen AT Bradley LM . Regulation of antigen-experienced T cells: Lessons from the quintessential memory marker CD44. Front Immunol. (2012) 3. doi: 10.3389/fimmu.2012.00023 22566907 PMC3342067

[19] Fu Q Li W Li S Zhao X Xie H Zhang X . CD44 facilitates adherence of Streptococcus equi subsp. zooepidemicus to LA-4 cells. Microb Pathog. (2019) 128:250–3. doi: 10.1016/j.micpath.2019.01.016 30639625

[20] Garay J Piazuelo MB Majumdar S Li L Trillo-Tinoco J Del Valle L . The homing receptor CD44 is involved in the progression of precancerous gastric lesions in patients infected with Helicobacter pylori and in development of mucous metaplasia in mice. Cancer Lett. (2016) 371:90–8. doi: 10.1016/j.canlet.2015.10.037 26639196 PMC4714604

[21] He X Shi X Puthiyakunnon S Zhang L Zeng Q Li Y . CD44-mediated monocyte transmigration across Cryptococcus neoformans-infected brain microvascular endothelial cells is enhanced by HIV-1 gp41-I90 ectodomain. J BioMed Sci. (2016) 23:28. doi: 10.1186/s12929-016-0247-2 26897523 PMC4761181

[22] Orian-Rousseau V . CD44 acts as a signaling platform controlling tumor progression and metastasis. Front Immunol. (2015) 6. doi: 10.3389/fimmu.2015.00154 25904917 PMC4389564

[23] McDonald B Kubes P . Interactions between CD44 and hyaluronan in leukocyte trafficking. Front Immunol. (2015) 6. doi: 10.3389/fimmu.2015.00068 25741341 PMC4330908

[24] Gruber JV Holtz R Riemer J . Hyaluronic acid (HA) stimulates the *in vitro* expression of CD44 proteins but not HAS1 proteins in normal human epidermal keratinocytes (NHEKs) and is HA molecular weight dependent. J Cosmet Dermatol. (2022) 21:1193–8. doi: 10.1111/jocd.14188 33908161

[25] Lee-Sayer SSM Dong Y Arif AA Olsson M Brown KL Johnson P . The where, when, how, and why of hyaluronan binding by immune cells. Front Immunol. (2015) 6. doi: 10.3389/fimmu.2015.00150 25926830 PMC4396519

[26] Radolf JD Deka RK Anand A Šmajs D Norgard MV Yang XF . Treponema pallidum, the syphilis spirochete: Making a living as a stealth pathogen. Nat Rev Microbiol. (2016) 14:744–59. doi: 10.1038/nrmicro.2016.141 27721440 PMC5106329

[27] Sinclair LV Finlay D Feijoo C Cornish GH Gray A Ager A . Phosphatidylinositol-3-OH kinase and nutrient-sensing mTOR pathways control T lymphocyte trafficking. Nat Immunol. (2008) 9:513–21. doi: 10.1038/ni.1603 18391955 PMC2857321

[28] Vassena L Giuliani E Koppensteiner H Bolduan S Schindler M Doria M . HIV-1 Nef and Vpu interfere with L-selectin (CD62L) cell surface expression to inhibit adhesion and signaling in infected CD4^+^ T lymphocytes. J Virol. (2015) 89:5687–700. doi: 10.1128/JVI.00611-15 25822027 PMC4442509

[29] Yang S Liu F Wang QJ Rosenberg SA Morgan RA . The shedding of CD62L (L-selectin) regulates the acquisition of lytic activity in human tumor reactive T lymphocytes. PloS One. (2011) 6:e22560. doi: 10.1371/journal.pone.0022560 21829468 PMC3145643

[30] Natalini A Simonetti S Favaretto G Peruzzi G Antonangeli F Santoni A . OMIP ‐079: Cell cycle of CD4^+^ and CD8^+^ naïve/memory T cell subsets, and of Treg cells from mouse spleen. Cytometry A. (2021) 99:1171–5. doi: 10.1002/cyto.a.24509 34668313 PMC9543383

[31] Hengel RL Thaker V Pavlick MV Metcalf JA Dennis G Yang J . Cutting edge: L-selectin (CD62L) expression distinguishes small resting memory CD4+ T cells that preferentially respond to recall antigen. J Immunol. (2003) 170:28–32. doi: 10.4049/jimmunol.170.1.28 12496379

[32] Jameson SC Masopust D . Understanding subset diversity in T cell memory. Immunity. (2018) 48:214–26. doi: 10.1016/j.immuni.2018.02.010 29466754 PMC5863745

[33] Van Faassen H Saldanha M Gilbertson D Dudani R Krishnan L Sad S . Reducing the stimulation of CD8+ T cells during infection with intracellular bacteria promotes differentiation primarily into a central (CD62LhighCD44high) subset. J Immunol. (2005) 174:5341–50. doi: 10.4049/jimmunol.174.9.5341 15843531

[34] Krull AC Shearer JK Gorden PJ Cooper VL Phillips GJ Plummer PJ . Deep sequencing analysis reveals temporal microbiota changes associated with development of bovine digital dermatitis. Infect Immun. (2014) 82:3359–73. doi: 10.1128/IAI.02077-14 24866801 PMC4136199

[35] Refaai W Ducatelle R Geldhof P Mihi B El-shair M Opsomer G . Digital dermatitis in cattle is associated with an excessive innate immune response triggered by the keratinocytes. BMC Vet Res. (2013) 9:193. doi: 10.1186/1746-6148-9-193 24090086 PMC3851557

[36] Evans NJ Brown JM Scholey R Murray RD Birtles RJ Hart CA . Differential inflammatory responses of bovine foot skin fibroblasts and keratinocytes to digital dermatitis treponemes. Vet Immunol Immunopathol. (2014) 161:12–20. doi: 10.1016/j.vetimm.2014.05.005 25022220

[37] Wherry EJ Barber DL Kaech SM Blattman JN Ahmed R . Antigen-independent memory CD8 T cells do not develop during chronic viral infection. Proc Natl Acad Sci. (2004) 101:16004–9. doi: 10.1073/pnas.0407192101 15505208 PMC524220

[38] Haining WN Neuberg DS Keczkemethy HL Evans JW Rivoli S Gelman R . Antigen-specific T-cell memory is preserved in children treated for acute lymphoblastic leukemia. Blood. (2005) 106:1749–54. doi: 10.1182/blood-2005-03-1082 15920008 PMC1895221

[39] Snell LM MacLeod BL Law JC Osokine I Elsaesser HJ Hezaveh K . CD8+ T cell priming in established chronic viral infection preferentially directs differentiation of memory-like cells for sustained immunity. Immunity. (2018) 49:678–694.e5. doi: 10.1016/j.immuni.2018.08.002 30314757 PMC8060917

[40] Croft M . Costimulation of T cells by OX40, 4-1BB, and CD27. Cytokine Growth Factor Rev. (2003) 14:265–73. doi: 10.1016/S1359-6101(03)00025-X 12787564

[41] Hendriks J Gravestein LA Tesselaar K Van Lier RAW Schumacher TNM Borst J . CD27 is required for generation and long-term maintenance of T cell immunity. Nat Immunol. (2000) 1:433–40. doi: 10.1038/80877 11062504

[42] Arens R Schepers K Nolte MA Van Oosterwijk MF Van Lier RAW Schumacher TNM . Tumor rejection induced by CD70-mediated quantitative and qualitative effects on effector CD8+ T cell formation. J Exp Med. (2004) 199:1595–605. doi: 10.1084/jem.20031111 15184507 PMC2211777

[43] Carrette F Surh CD . IL-7 signaling and CD127 receptor regulation in the control of T cell homeostasis. Semin Immunol. (2012) 24:209–17. doi: 10.1016/j.smim.2012.04.010 22551764 PMC3367861

[44] Bachmann MF Wolint P Schwarz K Oxenius A . Recall proliferation potential of memory CD8+ T cells and antiviral protection. J Immunol. (2005) 175:4677–85. doi: 10.4049/jimmunol.175.7.4677 16177115

[45] Mackall CL Fry TJ Gress RE . Harnessing the biology of IL-7 for therapeutic application. Nat Rev Immunol. (2011) 11:330–42. doi: 10.1038/nri2970 21508983 PMC7351348

[46] van Gisbergen KPJM Klarenbeek PL Kragten NAM Unger PPA Nieuwenhuis MBB Wensveen FM . The costimulatory molecule CD27 maintains clonally diverse CD8+ T cell responses of low antigen affinity to protect against viral variants. Immunity. (2011) 35:97–108. doi: 10.1016/j.immuni.2011.04.020 21763160

[47] De Colvenaer V Taveirne S Hamann J De Bruin AM De Smedt M Taghon T . Continuous CD27 triggering *in vivo* strongly reduces NK cell numbers. Eur J Immunol. (2010) 40:1107–17. doi: 10.1002/eji.200939251 20140903

[48] Ochsenbein AF Riddell SR Brown M Corey L Baerlocher GM Lansdorp PM . CD27 expression promotes long-term survival of functional effector–memory CD8 + cytotoxic T lymphocytes in HIV-infected patients. J Exp Med. (2004) 200:1407–17. doi: 10.1084/jem.20040717 15583014 PMC2211945

[49] Paiardini M Cervasi B Albrecht H Muthukumar A Dunham R Gordon S . Loss of CD127 expression defines an expansion of effector CD8+ T cells in HIV-infected individuals1. J Immunol. (2005) 174:2900–9. doi: 10.4049/jimmunol.174.5.2900 15728501

[50] Penaloza-MacMaster P Rasheed AU Iyer SS Yagita H Blazar BR Ahmed R . Opposing effects of CD70 costimulation during acute and chronic lymphocytic choriomeningitis virus infection of mice. J Virol. (2011) 85:6168–74. doi: 10.1128/JVI.02205-10 21507976 PMC3126534

[51] Borst J Hendriks J Xiao Y . CD27 and CD70 in T cell and B cell activation. Curr Opin Immunol. (2005) 17:275–81. doi: 10.1016/j.coi.2005.04.004 15886117

[52] Matter M Odermatt B Yagita H Nuoffer JM Ochsenbein AF . Elimination of chronic viral infection by blocking CD27 signaling. J Exp Med. (2006) 203:2145–55. doi: 10.1084/jem.20060651 16923852 PMC2118404

[53] Sakaguchi S Yamaguchi T Nomura T Ono M . Regulatory T cells and immune tolerance. Cell. (2008) 133:775–87. doi: 10.1016/j.cell.2008.05.009 18510923

[54] Rudensky AY . Regulatory T cells and foxp3. Immunol Rev. (2011) 241:260–8. doi: 10.1111/j.1600-065X.2011.01018.x 21488902 PMC3077798

[55] Belkaid Y . Regulatory T cells and infection: a dangerous necessity. Nat Rev Immunol. (2007) 7:875–88. doi: 10.1038/nri2189 17948021

[56] Rowe JH Ertelt JM Way SS . Foxp3^+^ regulatory T cells, immune stimulation and host defence against infection. Immunology. (2012) 136:1–10. doi: 10.1111/j.1365-2567.2011.03551.x 22211994 PMC3372751

[57] Babolin C Amedei A Ozoliņš D Žileviča A D’Elios MM De Bernard M . TpF1 from Treponema pallidum activates inflammasome and promotes the development of regulatory T cells. J Immunol. (2011) 187:1377–84. doi: 10.4049/jimmunol.1100615 21709157

[58] Lundgren A Suri-Payer E Enarsson K Svennerholm AM Lundin BS . Helicobacter pylori - specific CD4^+^ CD25^high^ regulatory T cells suppress memory T-cell responses to H. pylori in infected individuals. Infect Immun. (2003) 71:1755–62. doi: 10.1128/IAI.71.4.1755-1762.2003 12654789 PMC152046

[59] Berod L Puttur F Huehn J Sparwasser T . Tregs in infection and vaccinology: heroes or traitors? Microb Biotechnol. (2012) 5:260–9. doi: 10.1111/j.1751-7915.2011.00299.x 21951341 PMC3815786

[60] Biswas PS Bhagat G Pernis AB . IRF4 and its regulators: evolving insights into the pathogenesis of inflammatory arthritis? Immunol Rev. (2010) 233:79–96. doi: 10.1111/j.0105-2896.2009.00864.x 20192994 PMC2920730

[61] Harberts A Schmidt C Schmid J Reimers D Koch-Nolte F Mittrücker HW . Interferon regulatory factor 4 controls effector functions of CD8^+^ memory T cells. Proc Natl Acad Sci. (2021) 118:e2014553118. doi: 10.1073/pnas.2014553118 33859042 PMC8072204

[62] Man K Miasari M Shi W Xin A Henstridge DC Preston S . The transcription factor IRF4 is essential for TCR affinity–mediated metabolic programming and clonal expansion of T cells. Nat Immunol. (2013) 14:1155–65. doi: 10.1038/ni.2710 24056747

[63] Virgin HW Wherry EJ Ahmed R . Redefining chronic viral infection. Cell. (2009) 138:30–50. doi: 10.1016/j.cell.2009.06.036 19596234

[64] Wherry EJ Kurachi M . Molecular and cellular insights into T cell exhaustion. Nat Rev Immunol. (2015) 15:486–99. doi: 10.1038/nri3862 26205583 PMC4889009

[65] Hirsch T Neyens D Duhamel C Bayard A Vanhaver C Luyckx M . IRF4 impedes human CD8 T cell function and promotes cell proliferation and PD-1 expression. Cell Rep. (2024) 43:114401. doi: 10.1016/j.celrep.2024.114401 38943641

[66] Xia C Ya-dong G Jiong Y . Elevated interferon regulatory factor 4 levels in patients with allergic asthma. J Asthma. (2012) 49:441–9. doi: 10.3109/02770903.2012.674998 22515488

[67] Lu J Liang T Li P Yin Q . Regulatory effects of IRF4 on immune cells in the tumor microenvironment. Front Immunol. (2023) 14:1086803. doi: 10.3389/fimmu.2023.1086803 36814912 PMC9939821

[68] Kaminiów K Kiołbasa M Pastuszczak M . The significance of the cell-mediated host immune response in syphilis. Microorganisms. (2024) 12:2580. doi: 10.3390/microorganisms12122580 39770782 PMC11677580

[69] Urdahl KB Shafiani S Ernst JD . Initiation and regulation of T-cell responses in tuberculosis. Mucosal Immunol. (2011) 4:288–93. doi: 10.1038/mi.2011.10 21451503 PMC3206635

