# Supplementary material for: Altered splenic T-cell subpopulation dynamics in a murine model of bovine digital dermatitis-associated Treponema spp. infection
Source: Front Immunol. 2026 Jul 22;17:1744712. doi: 10.3389/fimmu.2026.1744712 (PMC13437327; doi:10.3389/fimmu.2026.1744712)
Supplement: Supplementary file 1 [file DataSheet1.pdf]

Supplementary Materials

Fig. S1. Representative flow-cytometry gating strategy for splenic lymphocyte subset analysis.

Fig. S2. Representative fluorescence minus one (FMO) controls used for marker-positive gate placement. (A) CD4, (B) CD8a, (C) CD44, (D) CD62L, (E) Foxp3, (F) CD127, (G) GATA3, (H) T-bet, (I) RORγT, (J) CD27 and (K) IRF4.

Fig. S3. Representative flow cytometry plots of splenic T-cell subsets following experimental infection with BDD-associated *Treponema* spp. Representative plots are shown for mice inoculated with control, Tpha, Tped, Tpes and Tcom at 1, 3, and 6 weeks post-infection. Panels show representative gating for (A) $CD4^+$, $CD44^+$ (B) $CD8^+$, $CD44^+$ (C) $CD4^+$, $CD44^+$, $CD62L^+$ (D) $CD8^+$, $CD44^+$, $CD62L^+$ (E) $CD8^+$, $CD44^+$, $CD27^+$, CD127- (F) $CD8^+$, $CD44^+$, $IRF4^+$ and (G–I) $CD4^+$ helper T-cell transcription factor subsets, including $Foxp3^+$, T-bet^+, $GATA3^+$, $ROR\gamma T^+$.

Table S1. Parent gates and denominators used for flow-cytometry subset analysis.

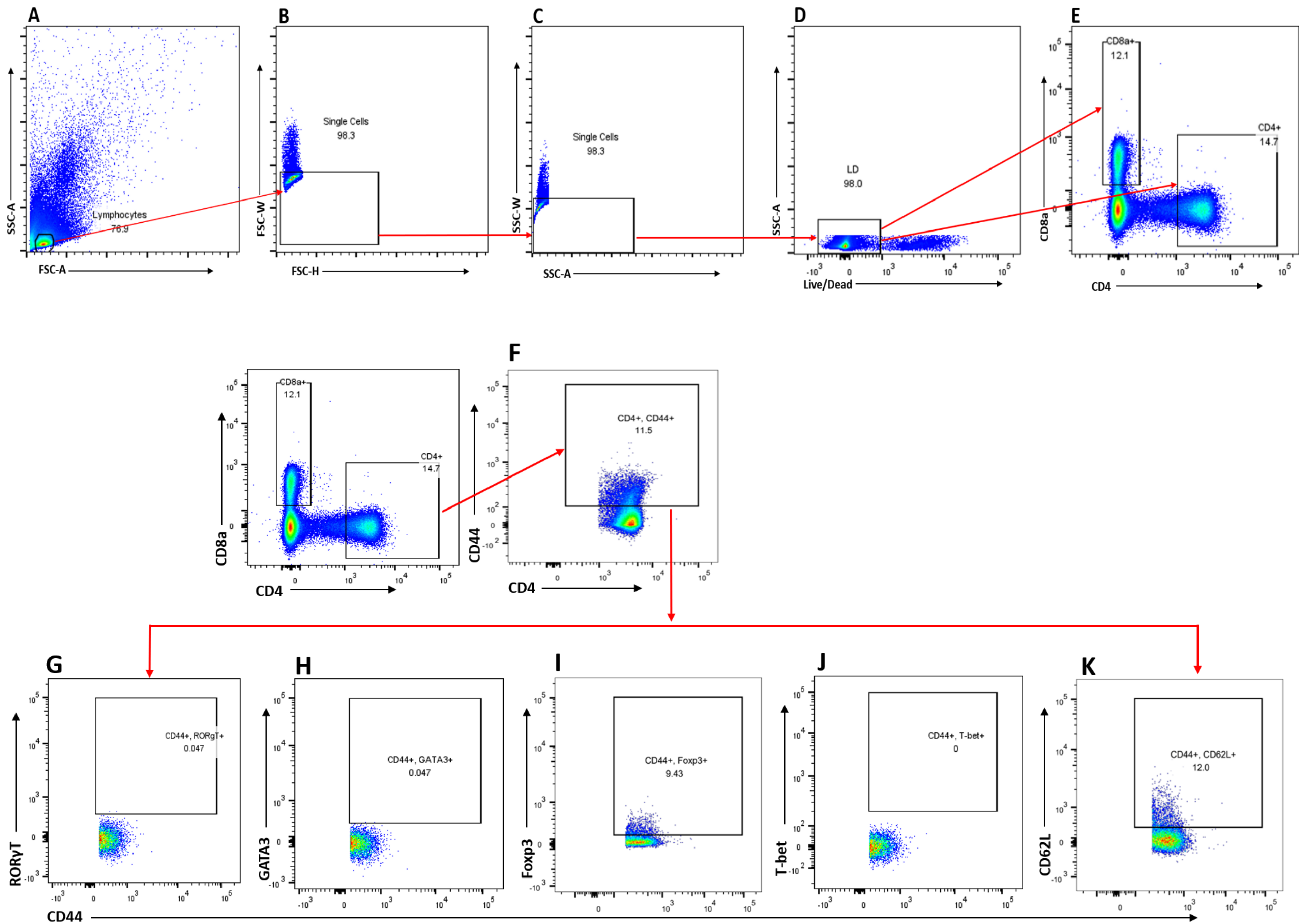

Fig. S1. Representative flow-cytometry gating strategy for splenic lymphocyte subset analysis.

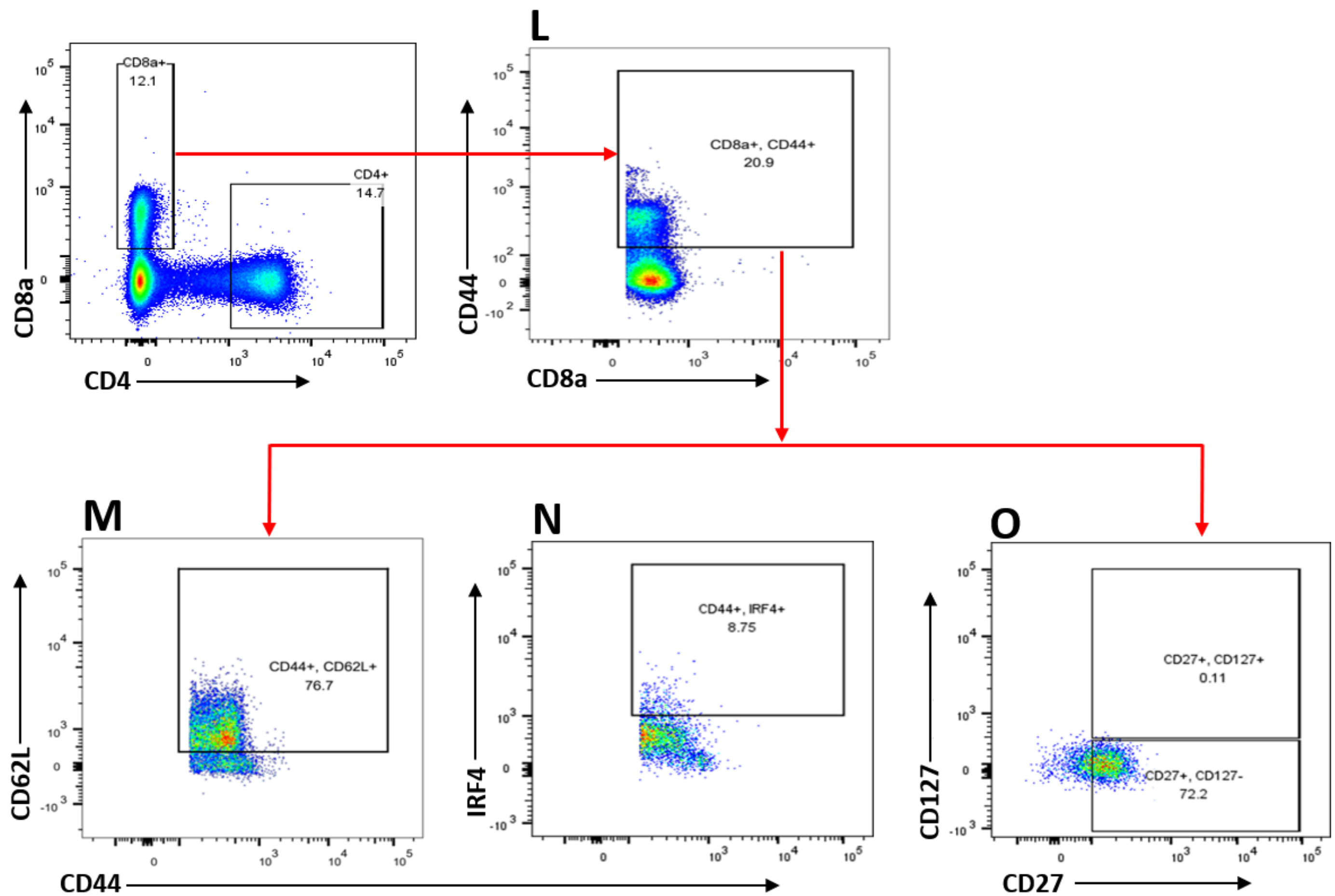

Fig. S1 continued. Representative flow-cytometry gating strategy for splenic lymphocyte subset analysis.

(A). CD4

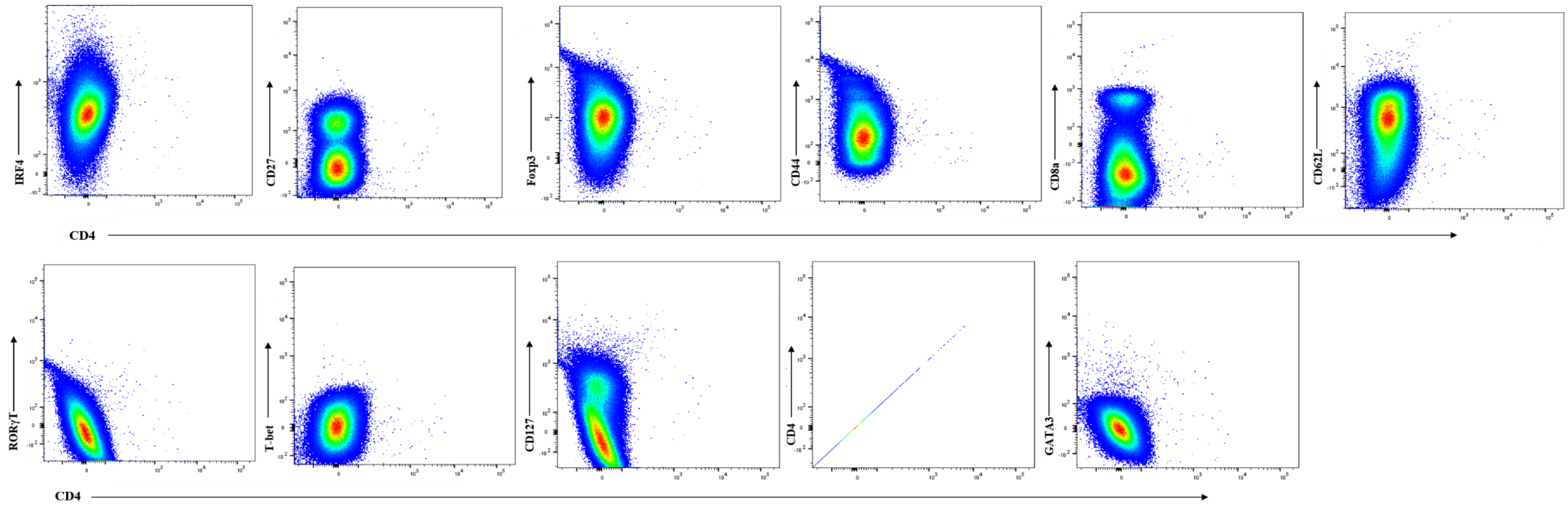

(B). CD8a

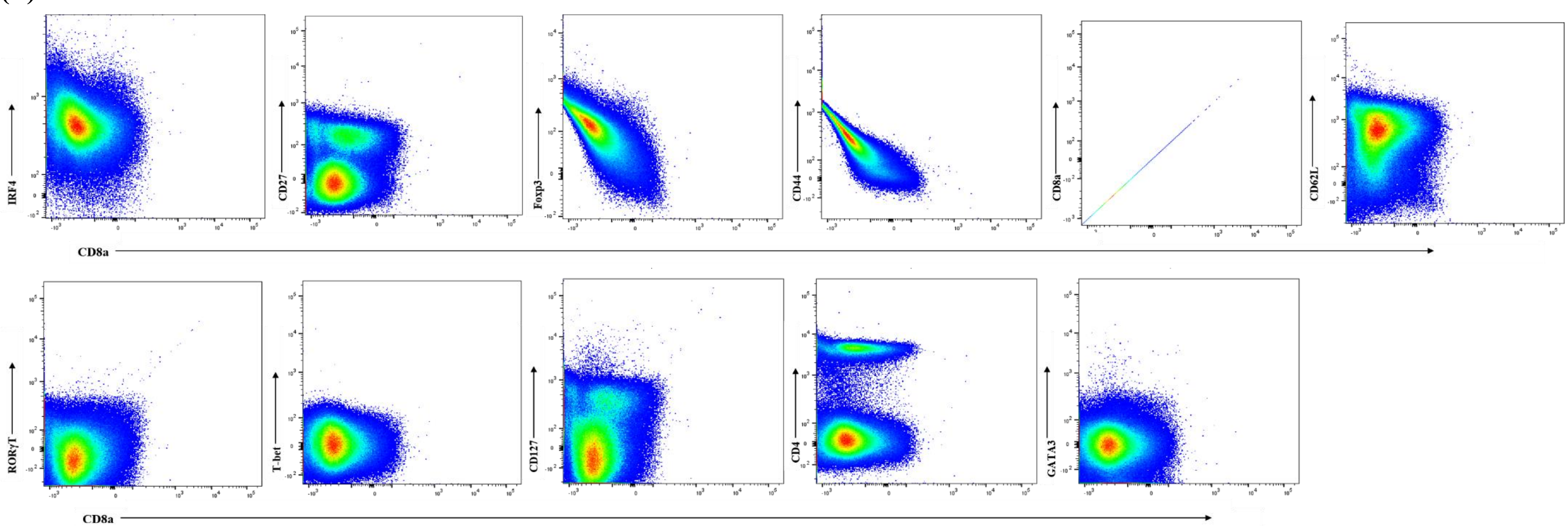

Fig. S2. Representative fluorescence minus one (FMO) controls used for marker-positive gate placement.

(C). CD44

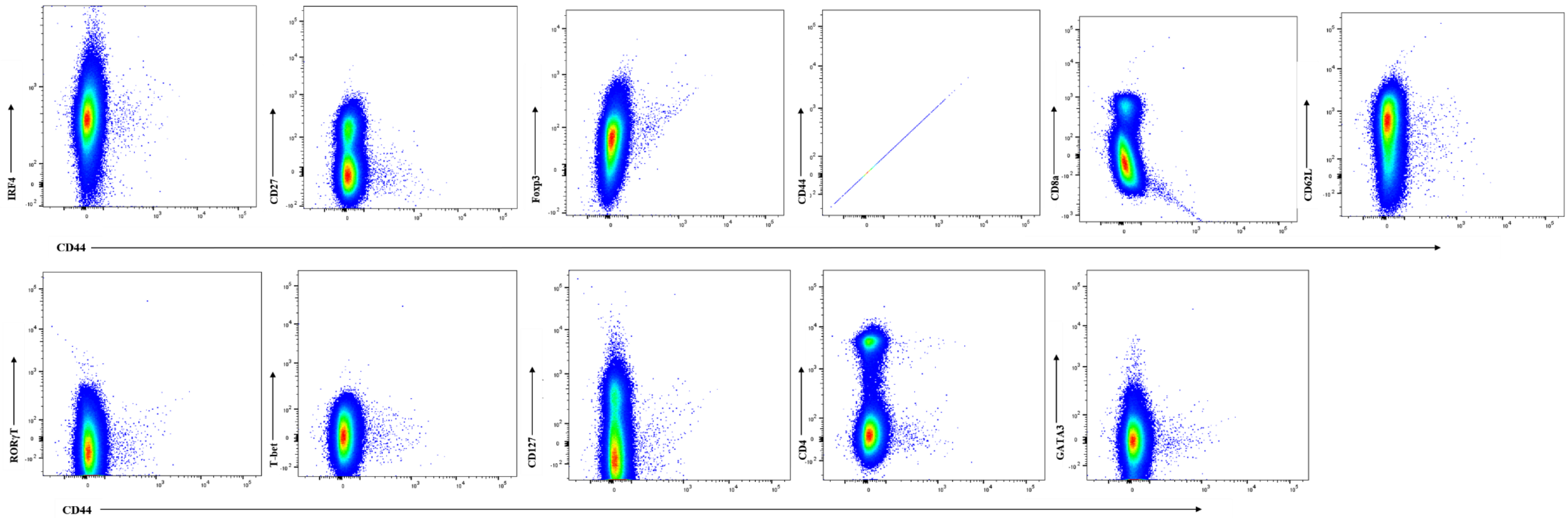

(D). CD62L

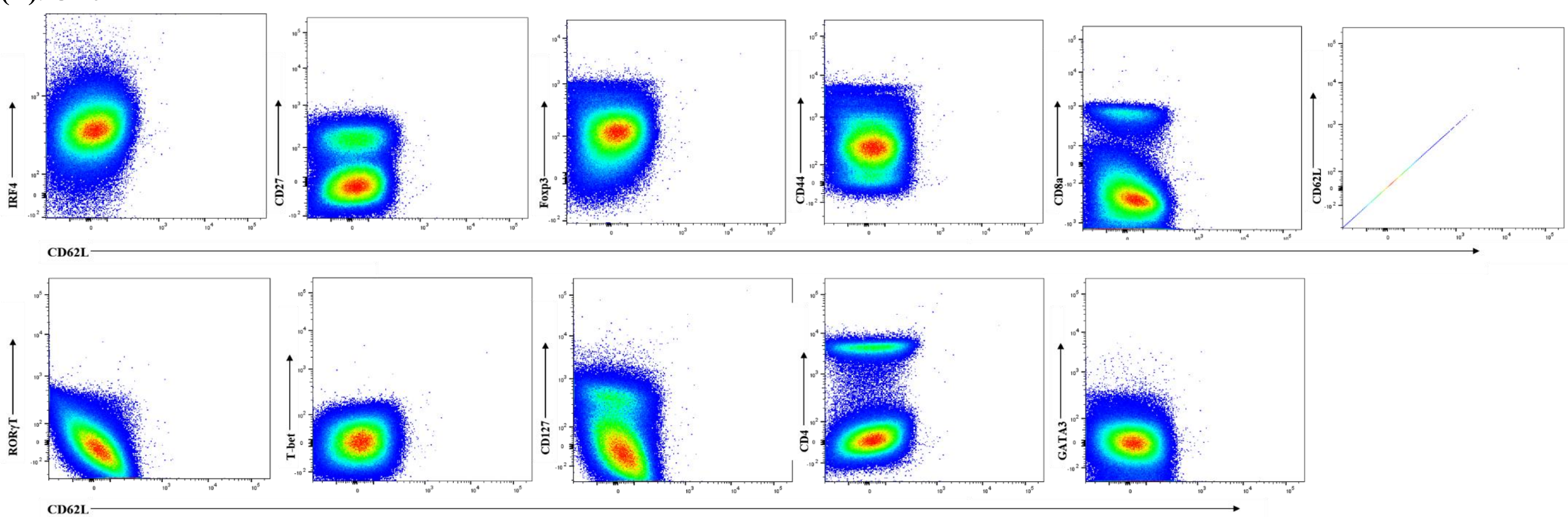

Fig. S2 continued. Representative fluorescence minus one (FMO) controls used for marker-positive gate placement.

(E). Foxp3

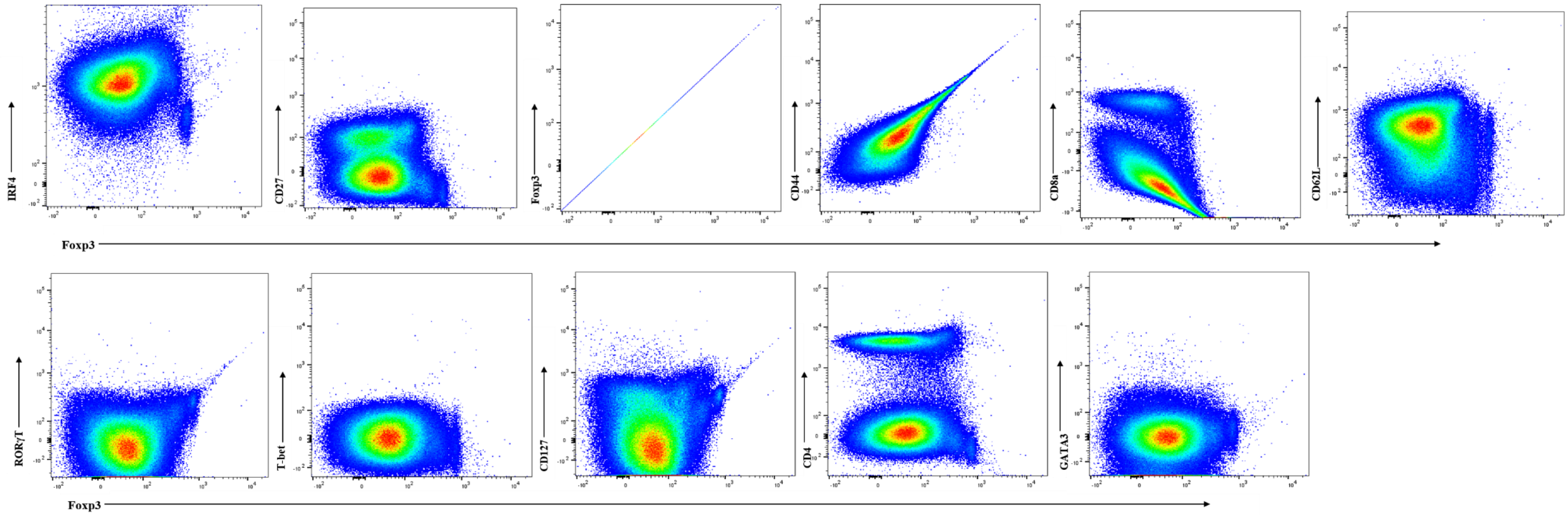

(F). CD127

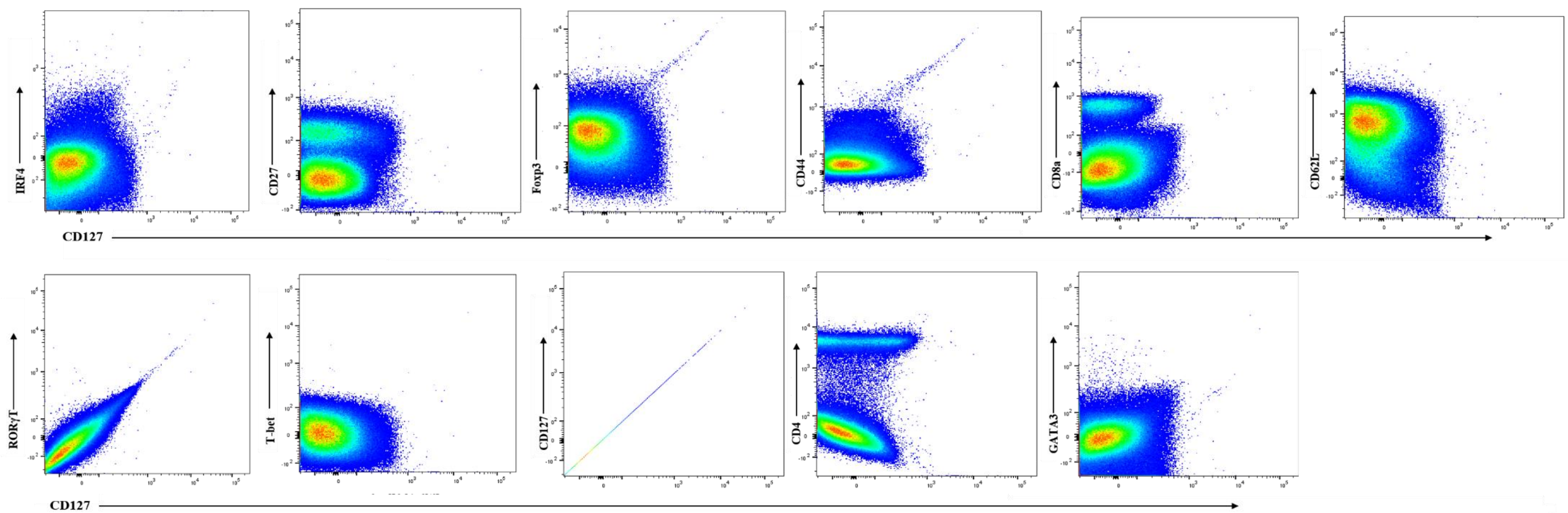

Fig. S2 continued. Representative fluorescence minus one (FMO) controls used for marker-positive gate placement.

(G). GATA3

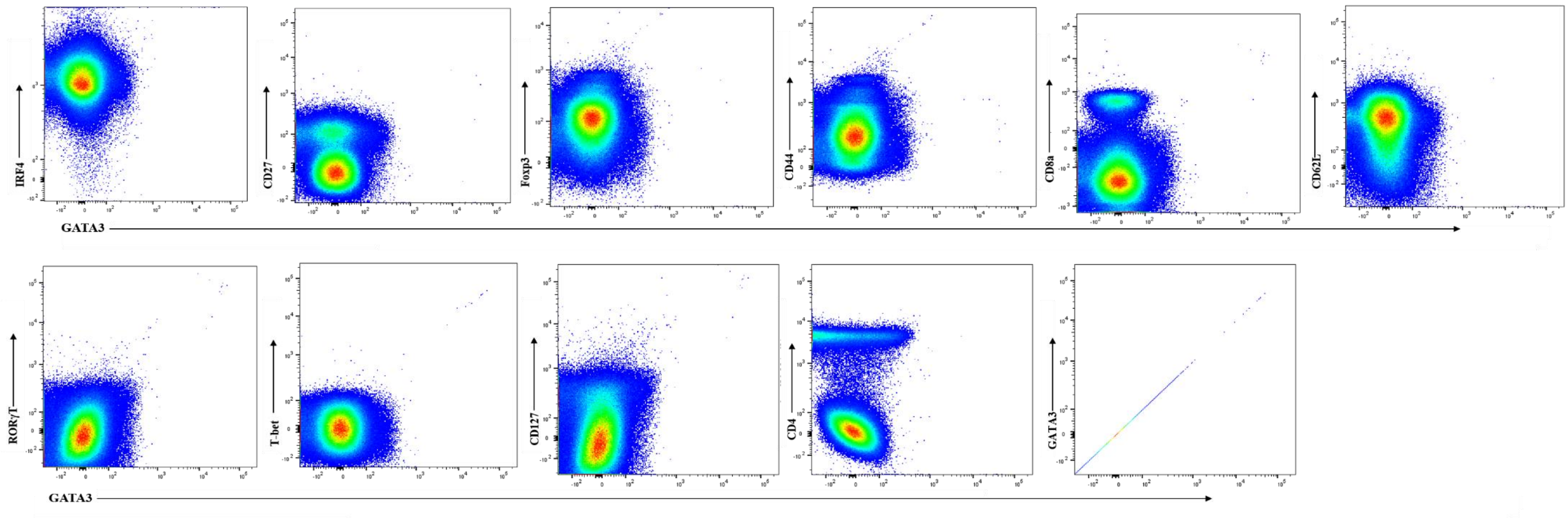

(H). T-bet

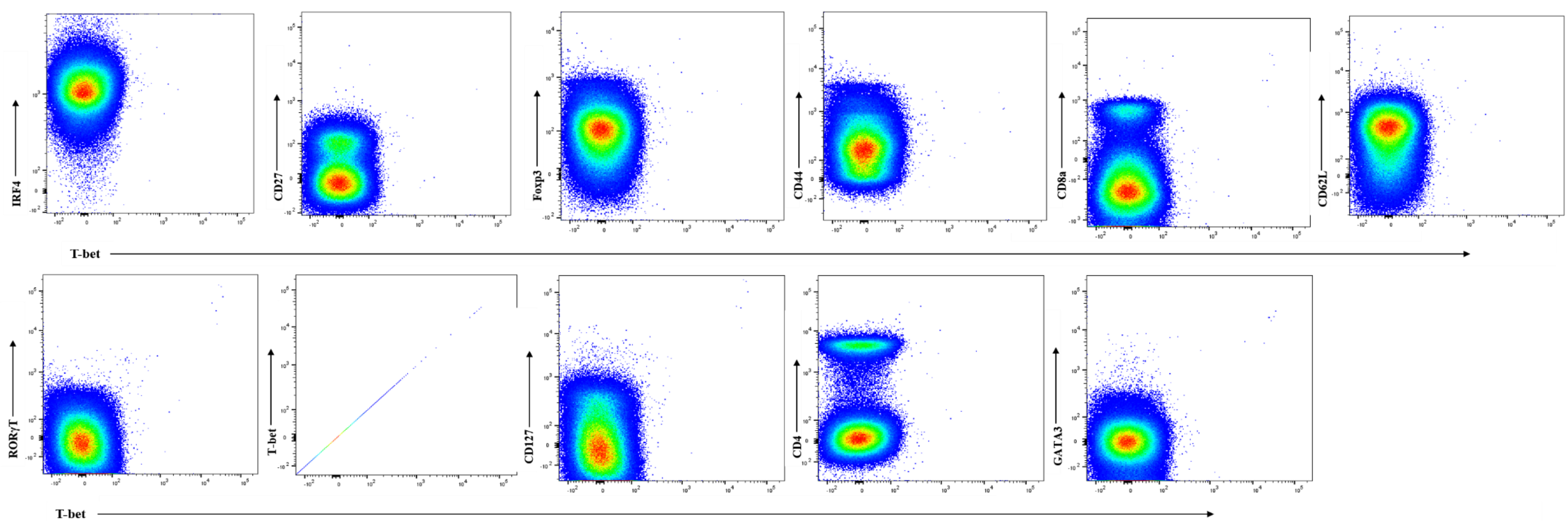

Fig. S2 continued. Representative fluorescence minus one (FMO) controls used for marker-positive gate placement.

(I). RORγT

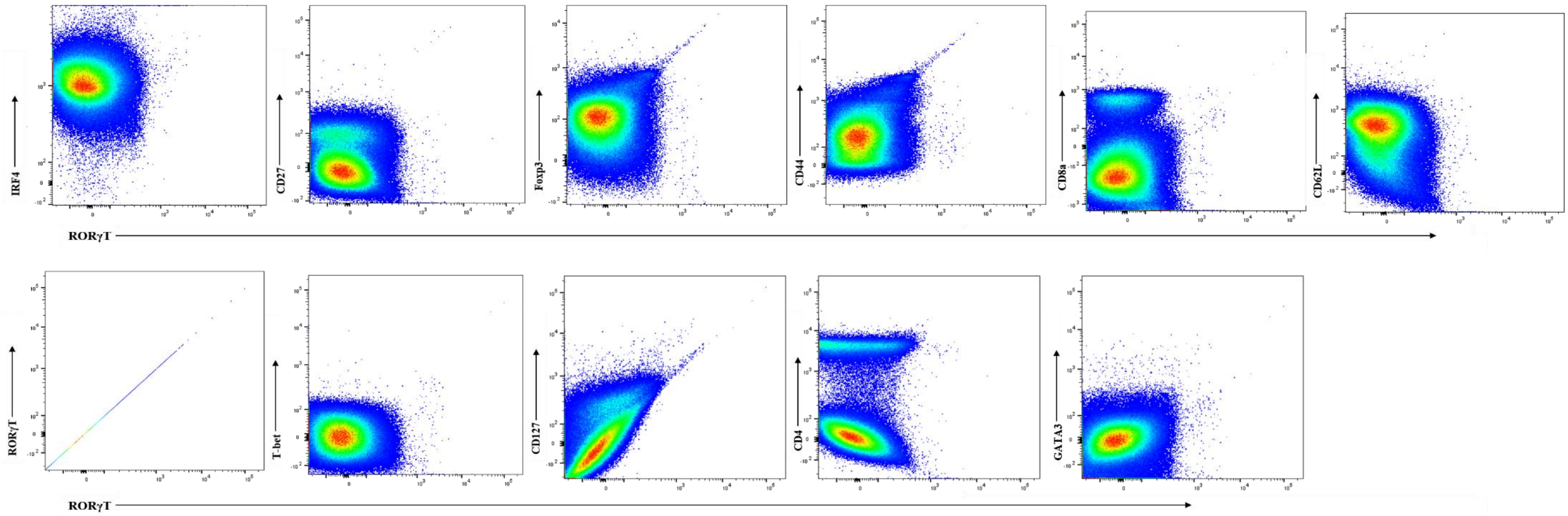

(J). CD27

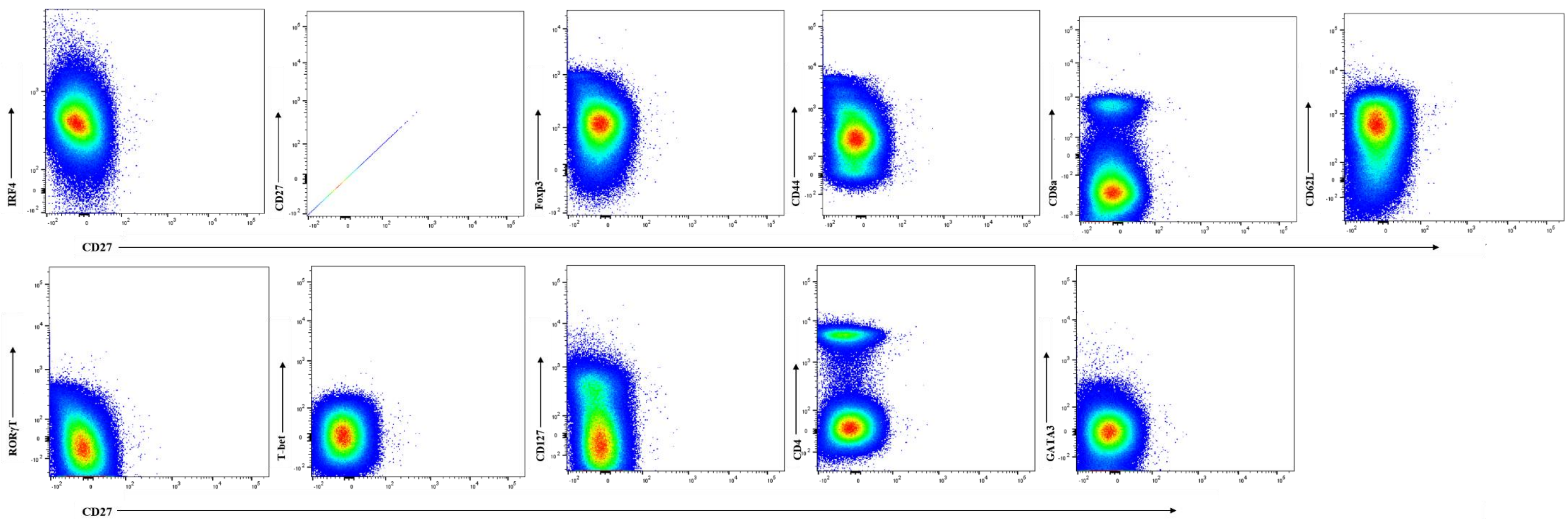

Fig. S2 continued. Representative fluorescence minus one (FMO) controls used for marker-positive gate placement.

(K). IRF4

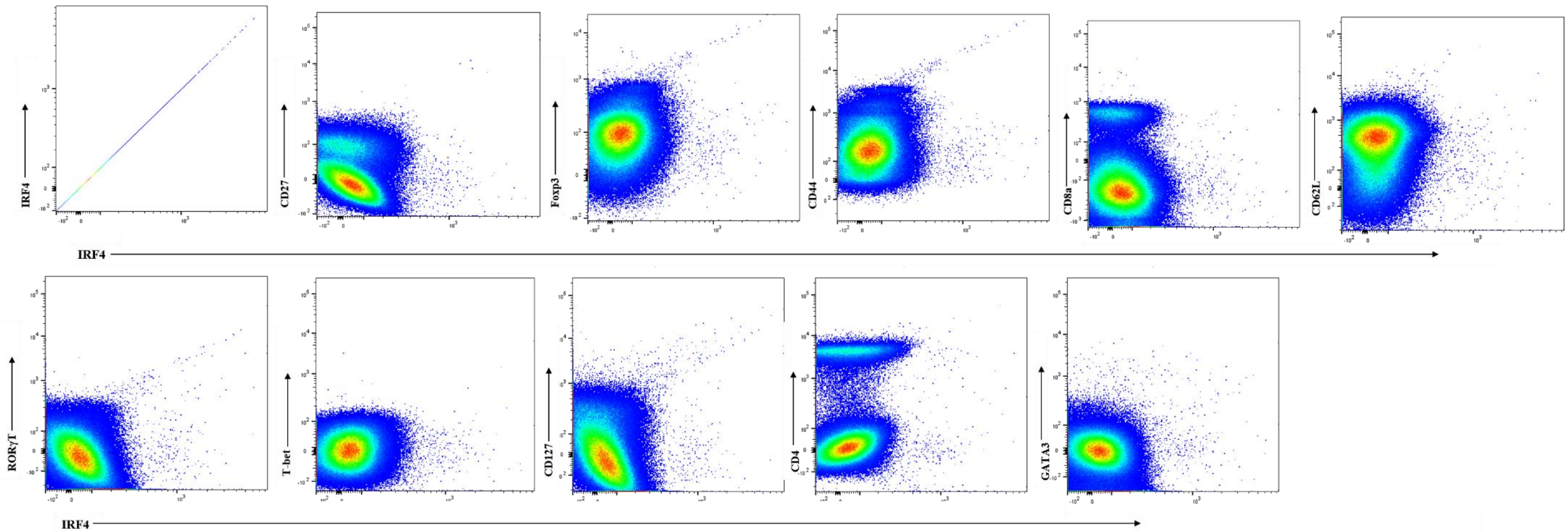

Fig. S2 continued. Representative fluorescence minus one (FMO) controls used for marker-positive gate placement.

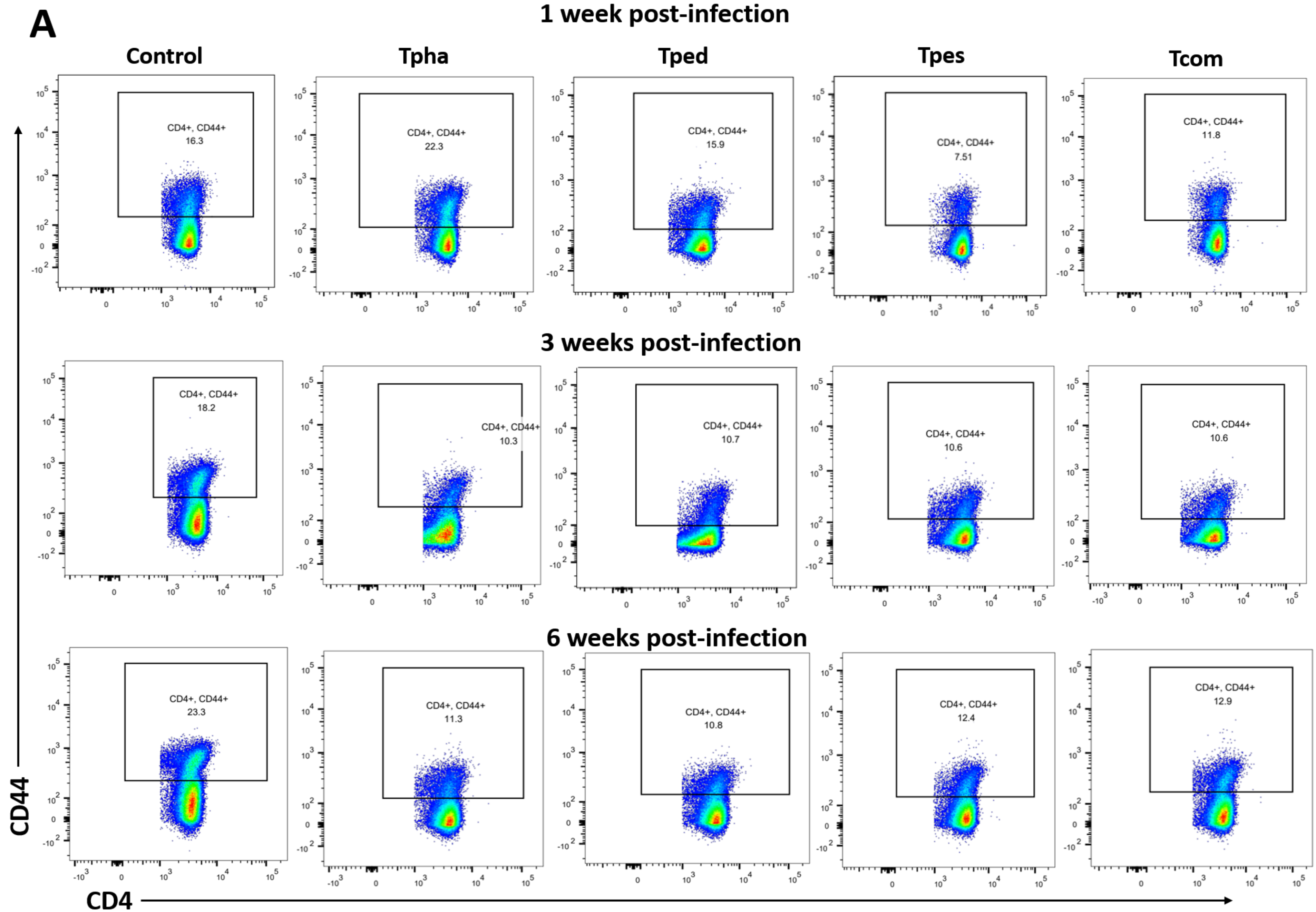
A
1 week post-infection
Control
Tpha
Tped
Tpes
Tcom
CD4+, CD44+
16.3
CD4+, CD44+
22.3
CD4+, CD44+
15.9
CD4+, CD44+
7.51
CD4+, CD44+
11.8
3 weeks post-infection
CD4+, CD44+
18.2
CD4+, CD44+
10.3
CD4+, CD44+
10.7
CD4+, CD44+
10.6
CD4+, CD44+
10.6
6 weeks post-infection
CD4+, CD44+
23.3
CD4+, CD44+
11.3
CD4+, CD44+
10.8
CD4+, CD44+
12.4
CD4+, CD44+
12.9
CD44
CD4

Fig. S3

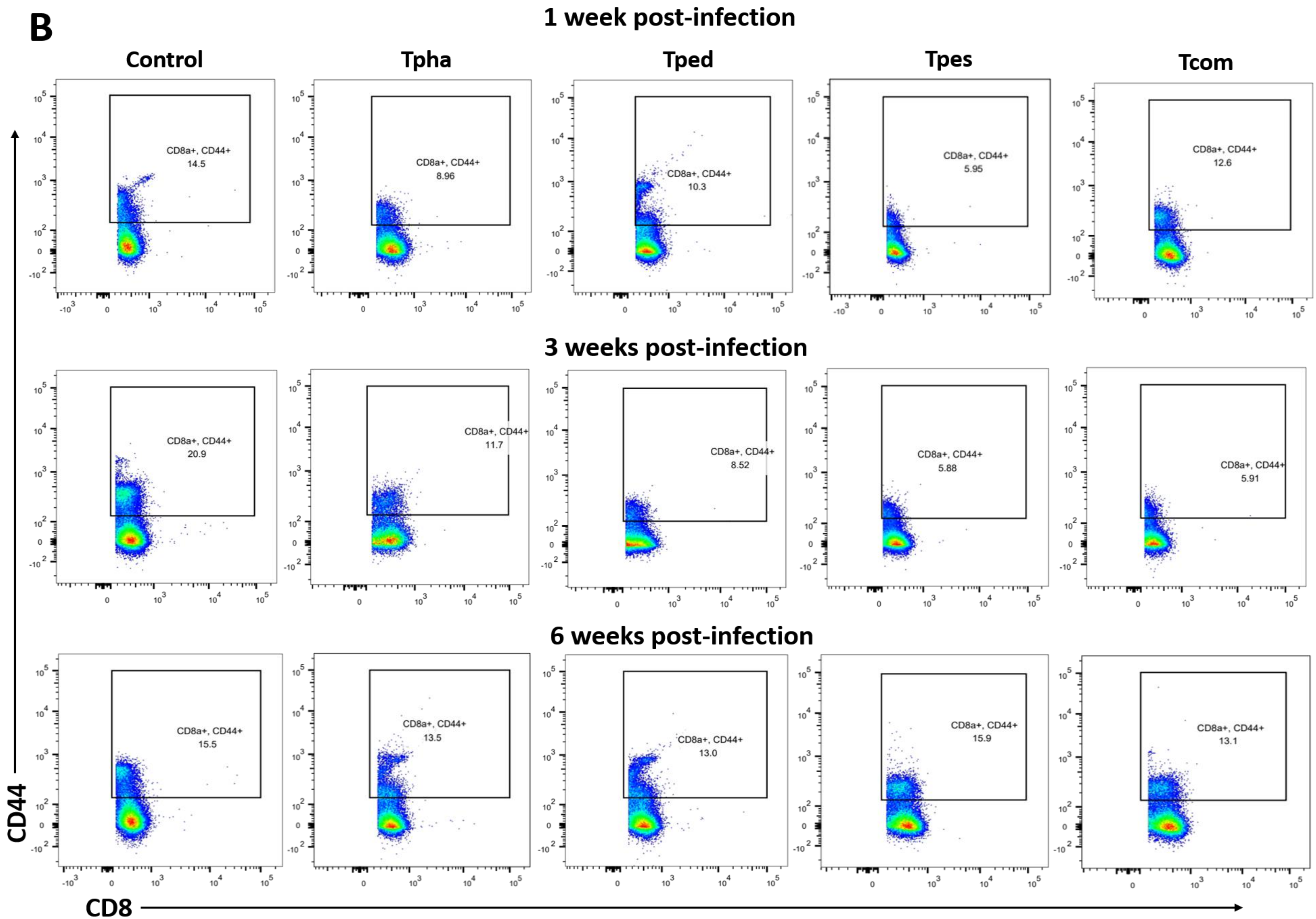

Fig. S3. continued

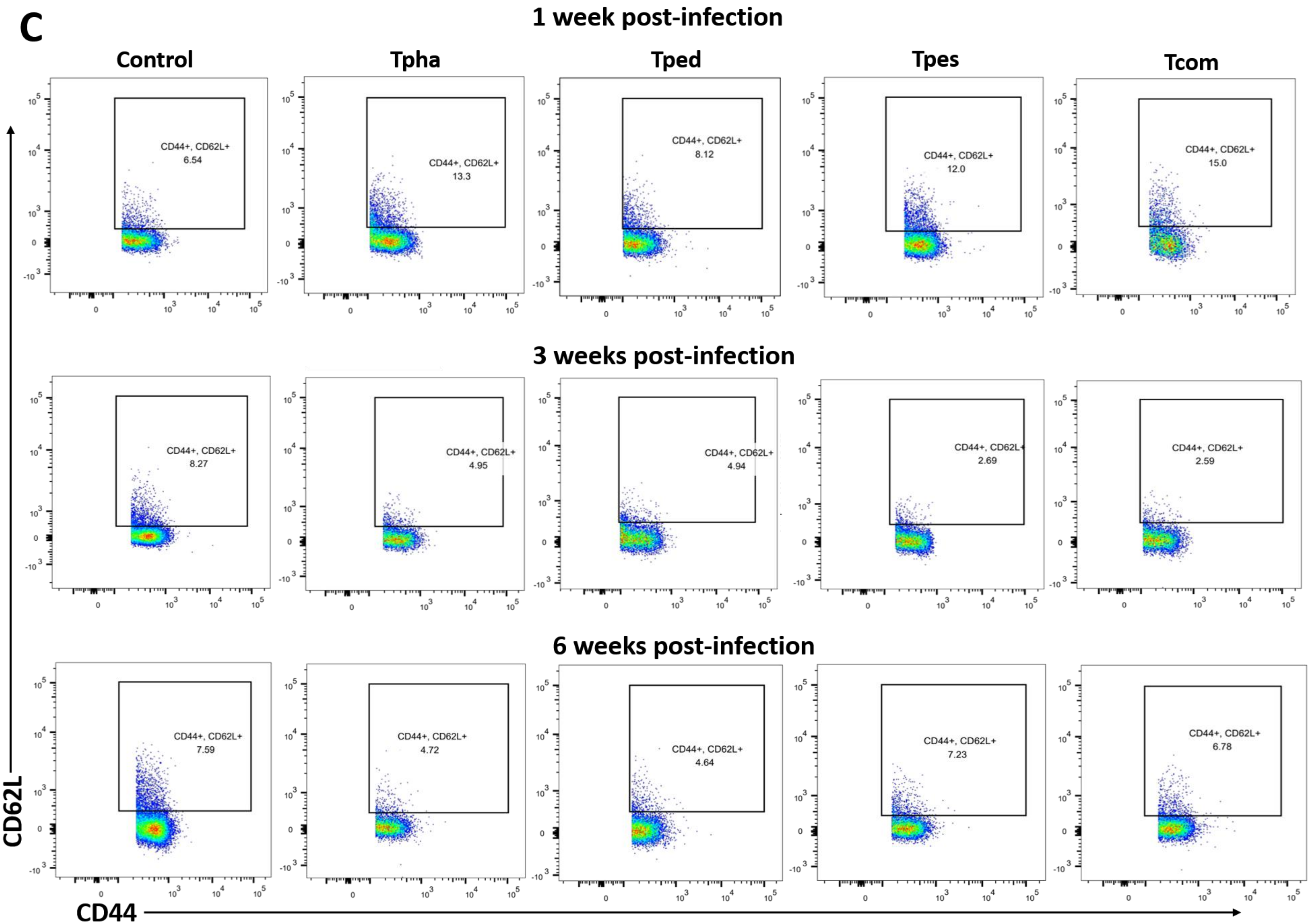

Fig. S3. continued

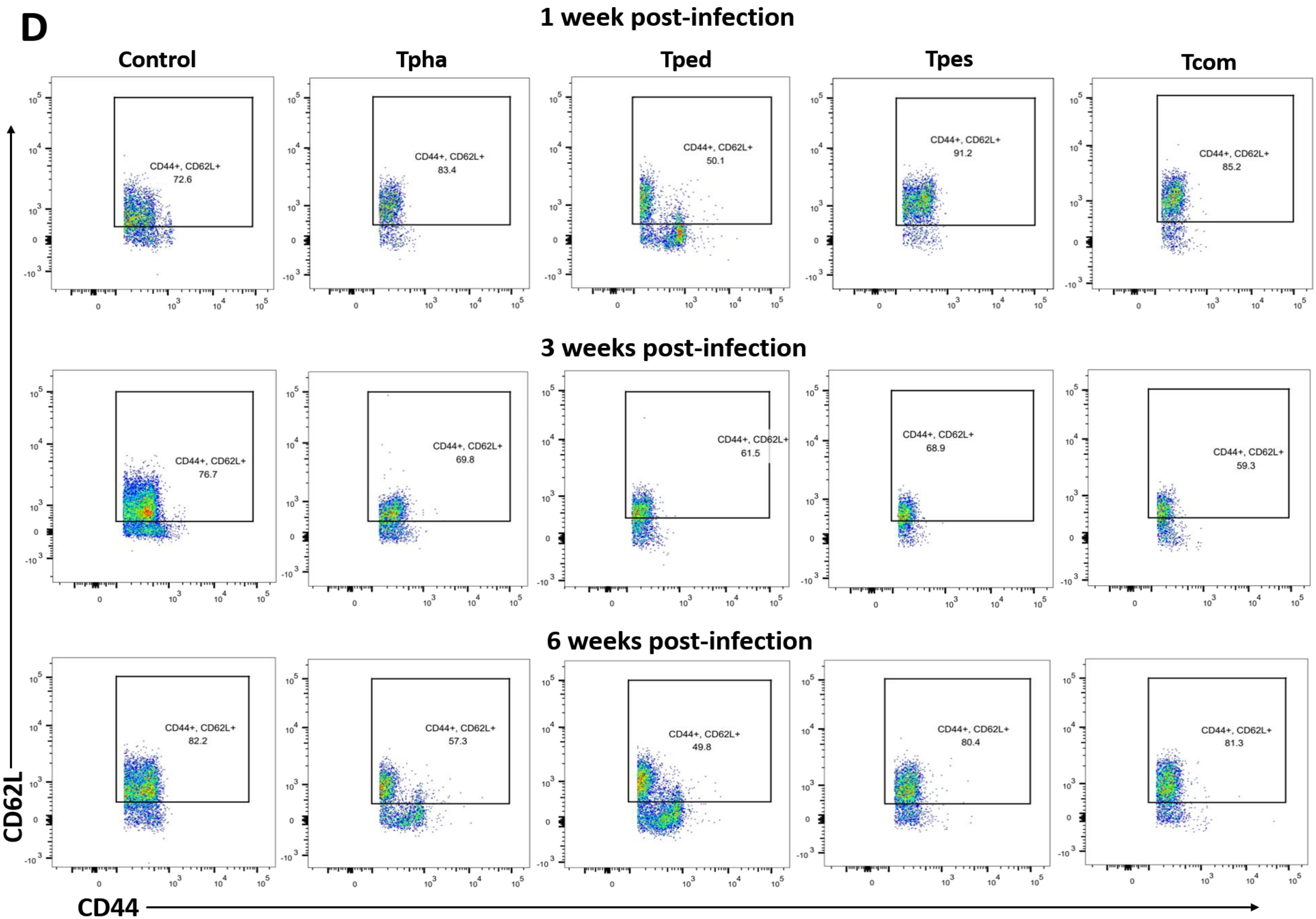

Fig. S3. continued

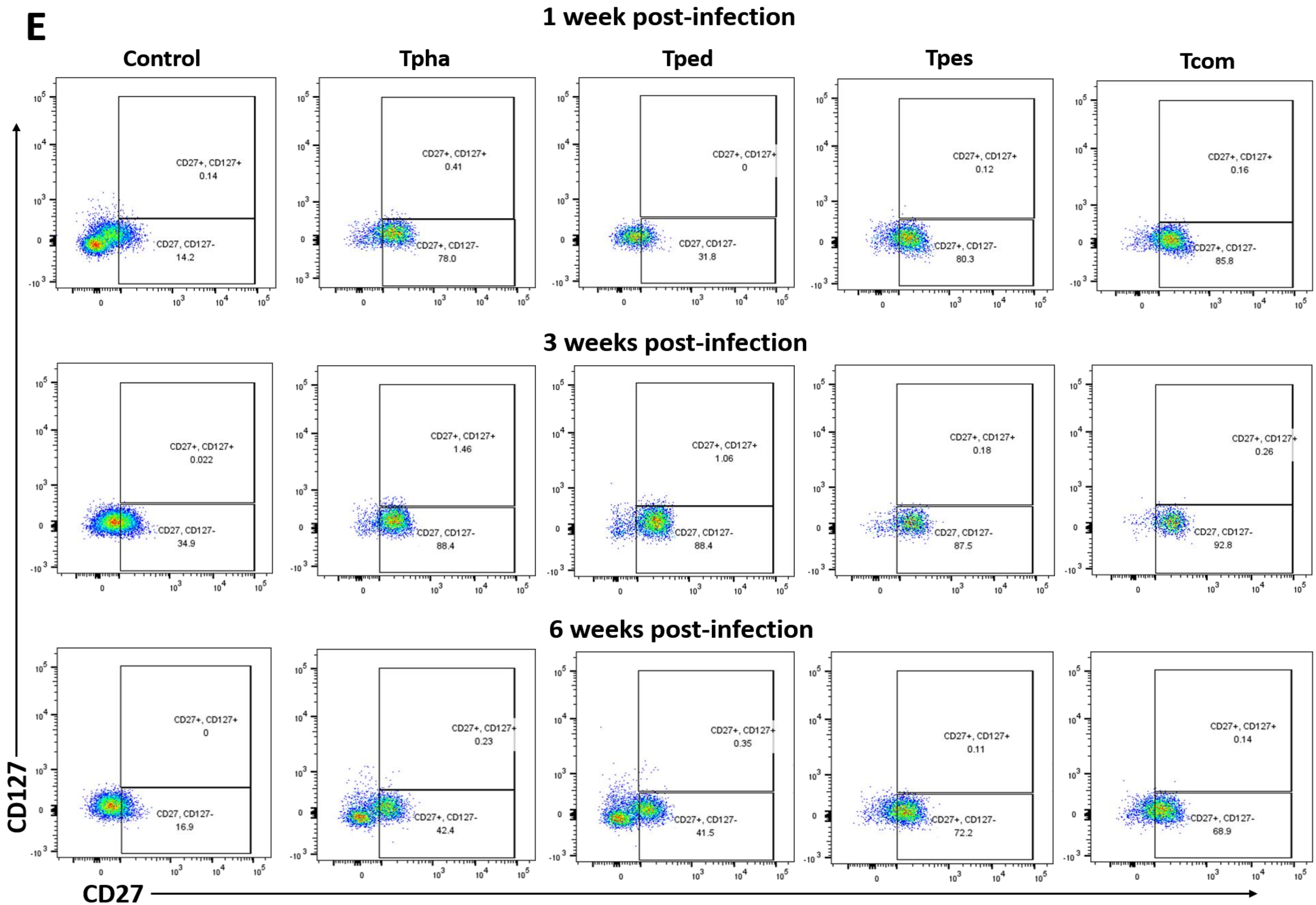

Fig. S3. continued

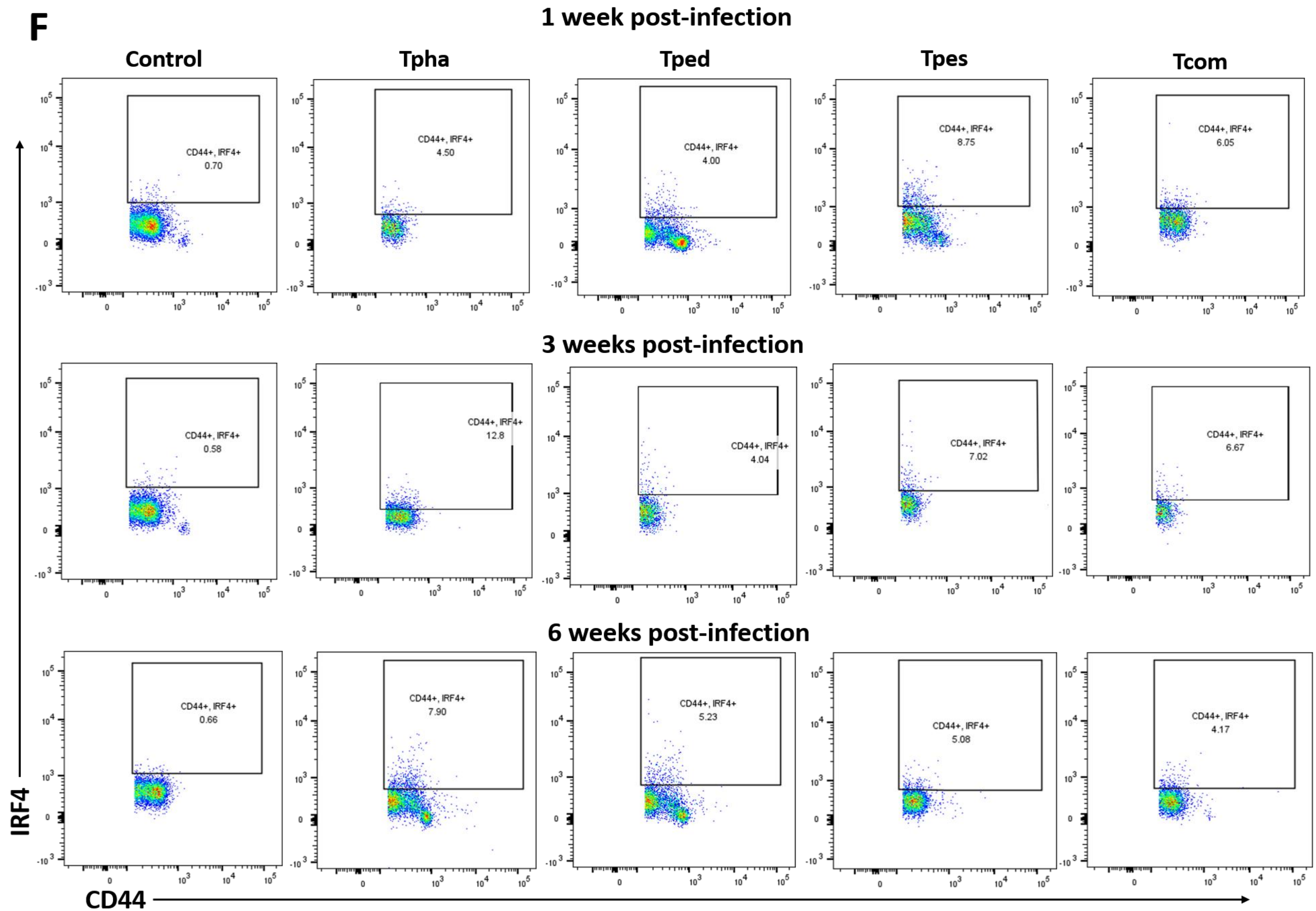

Fig. S3. continued

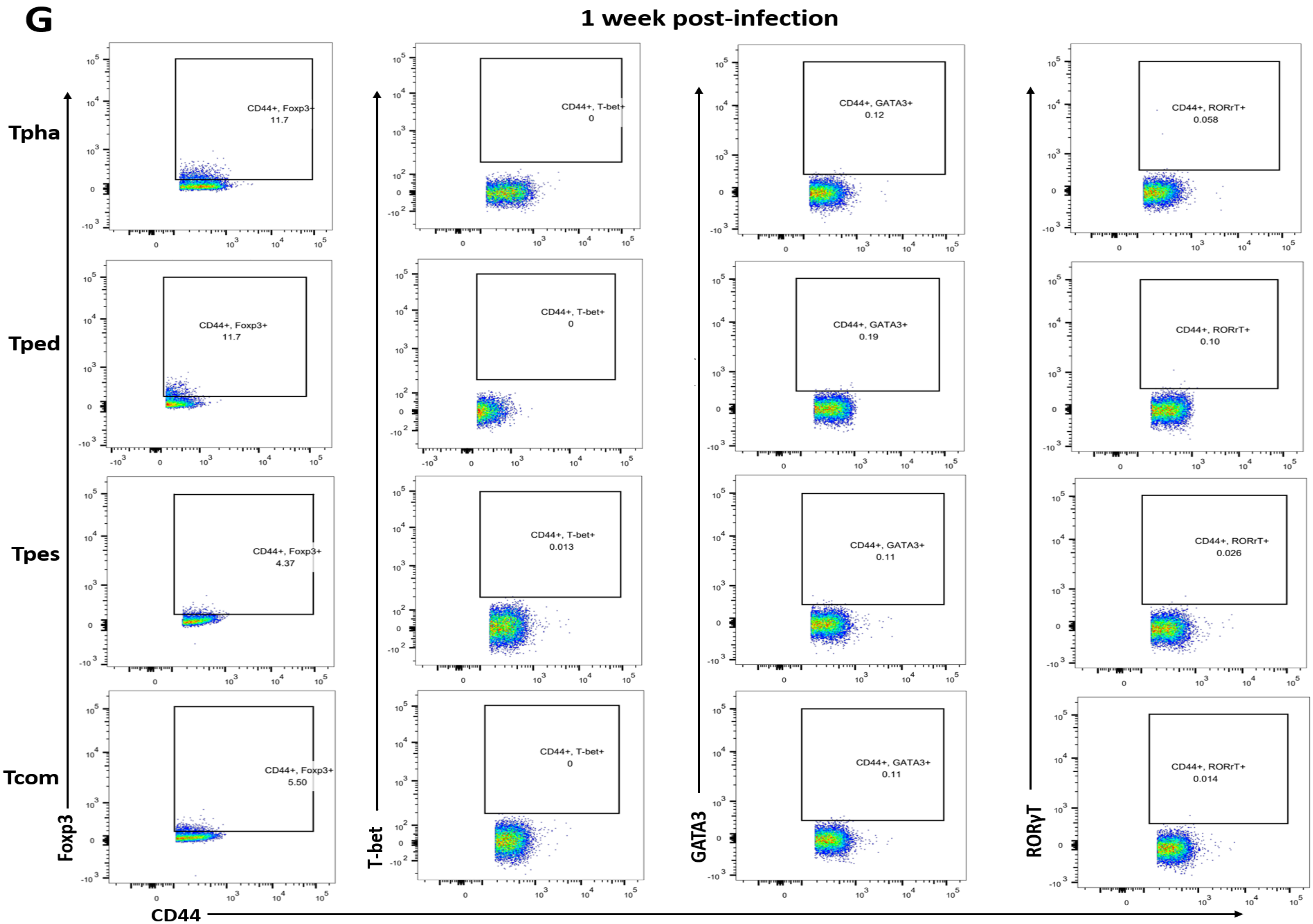

Fig. S3. continued

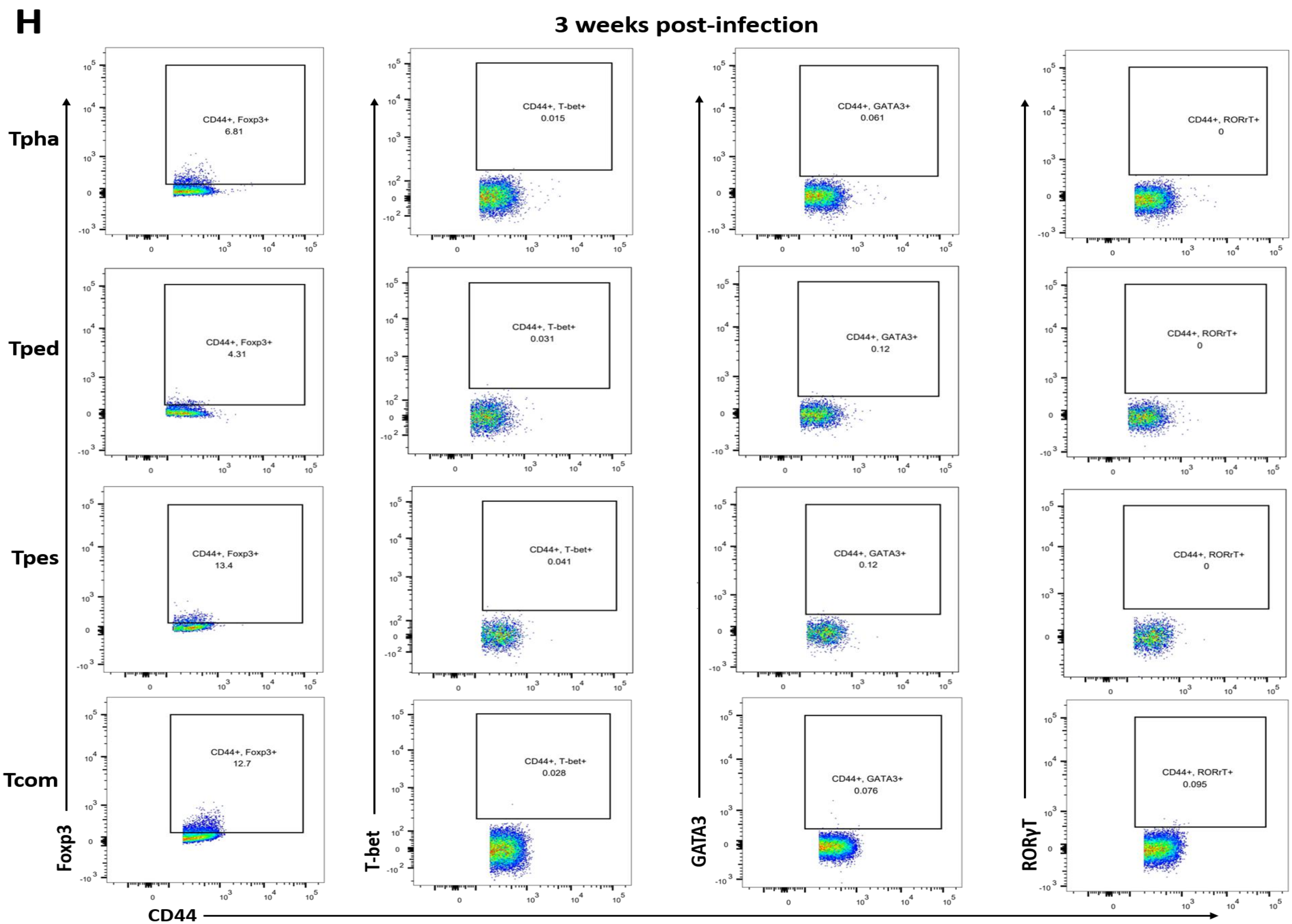

Fig. S3. continued

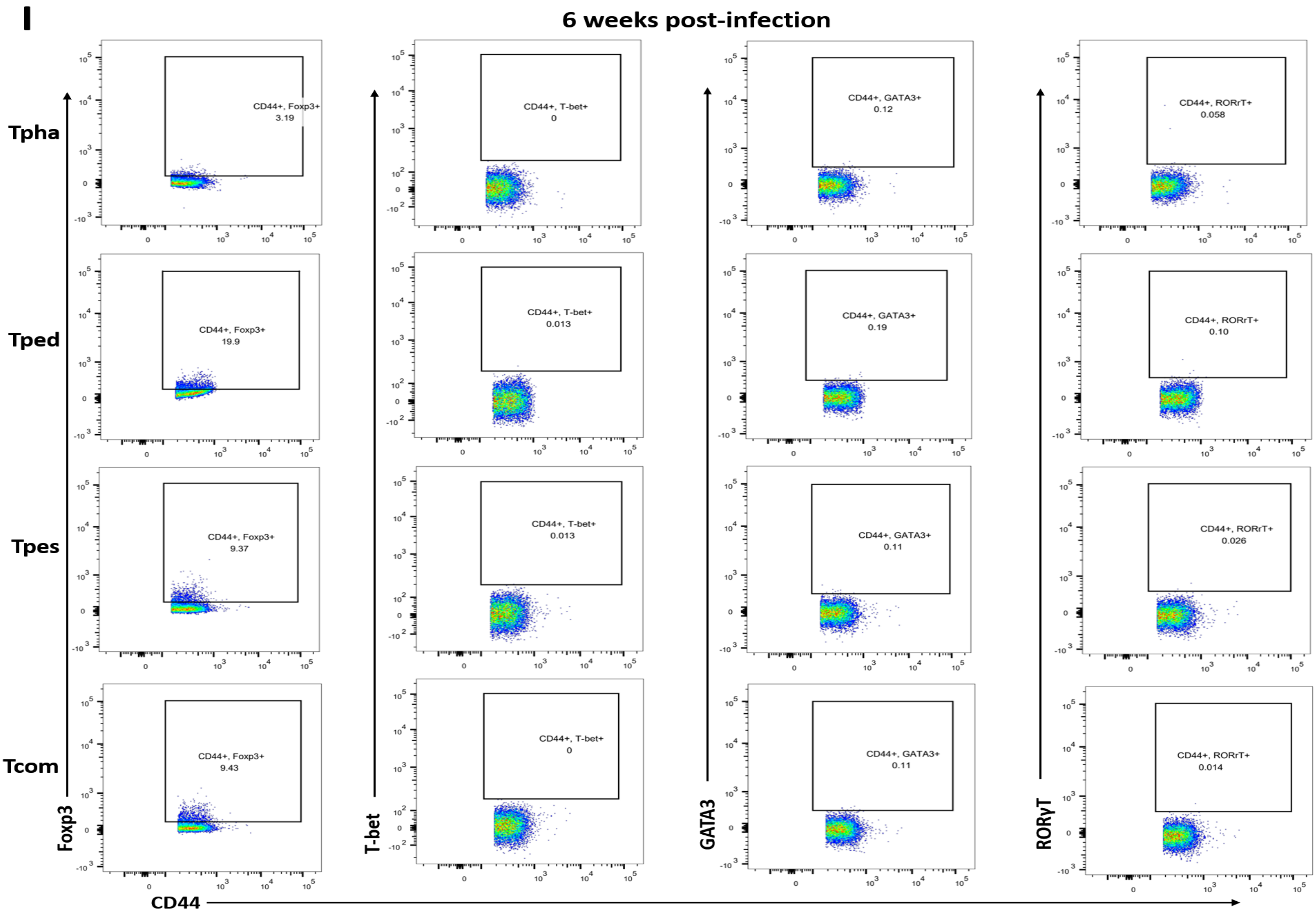

Fig. S3. continued

Table S1. Parent gates and denominators used for flow-cytometry subset analysis.

Reported subset	Parent gate / denominator
$CD4^{+}$, $CD44^{+}$	Live singlet $CD4^{+}$ lymphocytes
$CD8a^{+}$, $CD44^{+}$	Live singlet $CD8a^{+}$ lymphocytes
$CD4^{+}$, $CD44^{+}$, $CD62L^{+}$	Live singlet $CD4^{+}$, $CD44^{+}$ lymphocytes
$CD8a^{+}$, $CD44^{+}$, $CD62L^{+}$	Live singlet $CD8a^{+}$, $CD44^{+}$ lymphocytes
$CD8a^{+}$, $CD44^{+}$, $CD27^{+}$, $CD127^{-}$	Live singlet $CD8a^{+}$, $CD44^{+}$ lymphocytes
$CD8a^{+}$, $CD44^{+}$, $IRF4^{+}$	Live singlet $CD8a^{+}$, $CD44^{+}$ lymphocytes
$CD4^{+}$, $CD44^{+}$, $Foxp3^{+}$	Live singlet $CD4^{+}$, $CD44^{+}$ lymphocytes
$CD4^{+}$, $CD44^{+}$, T-bet^{+}	Live singlet $CD4^{+}$, $CD44^{+}$ lymphocytes
$CD4^{+}$, $CD44^{+}$, $GATA3^{+}$	Live singlet $CD4^{+}$, $CD44^{+}$ lymphocytes
$CD4^{+}$, $CD44^{+}$, $ROR\gamma t^{+}$	Live singlet $CD4^{+}$, $CD44^{+}$ lymphocytes
